# Extracellular G-quadruplexes and Z-DNA protect biofilms from DNase I, and G-quadruplexes form a DNAzyme with peroxidase activity

**DOI:** 10.1093/nar/gkae034

**Published:** 2024-01-31

**Authors:** Gabriel Antonio Salvador Minero, Andreas Møllebjerg, Celine Thiesen, Mikkel Illemann Johansen, Nis Pedersen Jørgensen, Victoria Birkedal, Daniel Erik Otzen, Rikke Louise Meyer

**Affiliations:** Interdisciplinary Nanoscience Center (iNANO), Aarhus University, Gustav Wieds Vej 14, 8000 Aarhus, Denmark; Interdisciplinary Nanoscience Center (iNANO), Aarhus University, Gustav Wieds Vej 14, 8000 Aarhus, Denmark; Interdisciplinary Nanoscience Center (iNANO), Aarhus University, Gustav Wieds Vej 14, 8000 Aarhus, Denmark; Department Infectious Diseases, Aarhus University Hospital, Palle Juul-Jensens bvld 99, 8200 Aarhus N, Denmark; Department Infectious Diseases, Aarhus University Hospital, Palle Juul-Jensens bvld 99, 8200 Aarhus N, Denmark; Interdisciplinary Nanoscience Center (iNANO), Aarhus University, Gustav Wieds Vej 14, 8000 Aarhus, Denmark; Department of Chemistry, Aarhus University, Langelandsgade 140, 8000 Aarhus, Denmark; Interdisciplinary Nanoscience Center (iNANO), Aarhus University, Gustav Wieds Vej 14, 8000 Aarhus, Denmark; Department of Molecular Biology and Genetics, Aarhus University, Universitetsbyen 81, 8000 Aarhus, Denmark; Interdisciplinary Nanoscience Center (iNANO), Aarhus University, Gustav Wieds Vej 14, 8000 Aarhus, Denmark; Department of Biology, Aarhus University, Ny Munkegade 114, 8000 Aarhus, Denmark

## Abstract

Many bacteria form biofilms to protect themselves from predators or stressful environmental conditions. In the biofilm, bacteria are embedded in a protective extracellular matrix composed of polysaccharides, proteins and extracellular DNA (eDNA). eDNA most often is released from lysed bacteria or host mammalian cells, and it is the only matrix component most biofilms appear to have in common. However, little is known about the form DNA takes in the extracellular space, and how different non-canonical DNA structures such as Z-DNA or G-quadruplexes might contribute to its function in the biofilm. The aim of this study was to determine if non-canonical DNA structures form in eDNA-rich staphylococcal biofilms, and if these structures protect the biofilm from degradation by nucleases. We grew *Staphylococcus epidermidis* biofilms in laboratory media supplemented with hemin and NaCl to stabilize secondary DNA structures and visualized their location by immunolabelling and fluorescence microscopy. We furthermore visualized the macroscopic biofilm structure by optical coherence tomography. We developed assays to quantify degradation of Z-DNA and G-quadruplex DNA oligos by different nucleases, and subsequently investigated how these enzymes affected eDNA in the biofilms.

Z-DNA and G-quadruplex DNA were abundant in the biofilm matrix, and were often present in a web-like structures. *In vitro*, the structures did not form in the absence of NaCl or mechanical shaking during biofilm growth, or in bacterial strains deficient in eDNA or exopolysaccharide production. We thus infer that eDNA and polysaccharides interact, leading to non-canonical DNA structures under mechanical stress when stabilized by salt. We also confirmed that G-quadruplex DNA and Z-DNA was present in biofilms from infected implants in a murine implant-associated osteomyelitis model. Mammalian DNase I lacked activity against Z-DNA and G-quadruplex DNA, while Micrococcal nuclease could degrade G-quadruplex DNA and S1 Aspergillus nuclease could degrade Z-DNA. Micrococcal nuclease, which originates from *Staphylococcus aureus*, may thus be key for dispersal of biofilm in staphylococci. In addition to its structural role, we show for the first time that the eDNA in biofilms forms a DNAzyme with peroxidase-like activity in the presence of hemin. While peroxidases are part of host defenses against pathogens, we now show that biofilms can possess intrinsic peroxidase activity in the extracellular matrix.

## Graphical Abstract

**Graphical Abstract:**
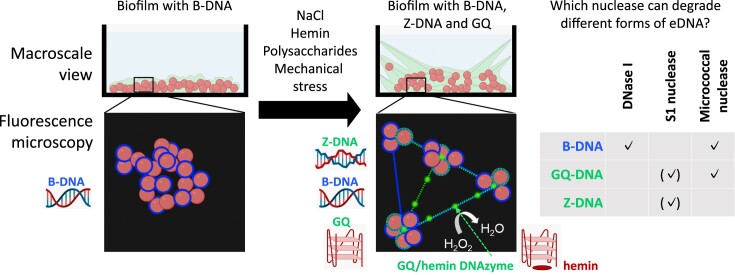

## Introduction

The most problematic bacterial infections today are those that involve biofilms: a multicellular community of microorganisms encased in a shared extracellular matrix. Biofilms consisting of pathogens are typically associated with implanted medical devices, and these are becoming a significant burden to the healthcare system as 25% of hospital-acquired infections are device-related (1). Biofilm formation is an important virulence factor, as it protects bacteria from the immune system and antibiotic therapy.

The biofilm matrix consists of polysaccharides, proteins, lipids and extracellular DNA (eDNA). While some bacteria produce toxins and several other virulence factors, the commensal skin bacterium *Staphylococcus epidermidis* relies primarily on biofilm formation to support its existence in the human body. Thanks to its ability to form a biofilm, *S. epidermidis* is a major cause of catheter-related bloodstream infections (2) and serious implant-associated infections such as prosthetic valve endocarditis (3), cardiac implantable electronic device infection (4) and prosthetic joint infection (5,6). Due to high rates of resistance to several classes of antimicrobials, treatment failure is high in prosthetic joint infections (6).

Among the mechanisms for biofilm formation, *S. epidermidis* uses eDNA, poly-*N*-acetylglucosamine (PNAG), and a plethora of cell wall-anchored proteins that are used for attachment to the host, and other molecules (7). One third of clinical isolates from implant-associated infections lack the genes for polysaccharide production (8). In contrast, most clinical isolates encode a giant 10 kDa surface protein, Embp (9), which is co-regulated with release of eDNA (7). eDNA was found in biofilm infections by *S. epidermidis* (10–12) and it is a critical component for establishment of biofilm, as removal of eDNA prevents biofilm formation by *S. epidermidis* (11) and many other bacterial species (13,14).

Efforts to disperse biofilms or facilitate the penetration of antibiotics (15) have long focused on eDNA as a prime target for enzymatic degradation of the biofilm matrix. Frustratingly, mammalian DNase I treatment only prevents formation of biofilms, while it is ineffective at breaking up mature biofilms (14) or biofilms obtained in media with high ionic strength (16). Until recently, it was assumed that eDNA exists primarily in its canonical right-handed B-form. However, Buzzo *et al.* reported on formation of the left-handed Z-DNA in various species of biofilms *in vitro* as well as *in vivo* (17). Moreover, Seviour *et al.* identified both G-quadruplex (GQ-) DNA and RNA in *Pseudomonas aeruginosa* biofilms (18,19). These non-canonical DNA structures are resistant to degradation by DNase I (20), and thus may explain why this enzyme fails to disperse biofilms.

In the cell, Z-DNA is found in supercoiled bacterial genomes or plasmids. Under physiological conditions, Z-DNA forms in (GC)_*n*_ sequences of *n* ≥ 8, while shorter (GC)_n_ sequences are in dynamic equilibrium between B- and Z-DNA conformations (21). *In vitro*, Z-DNA formation from linear non-supercoiled DNA oligos requires high salinity (up to 4.5 M NaCl) (22), polycationic molecules such as polyamines (e.g. spermine) (23), or cationic metalloporphyrins (24,25). Furthermore, different chemical modifications of nucleobases such as methylation or protonation of cytosine (26,27), or bromination or oxidation of guanine (28) can promote formation/stabilization of Z-DNA. It remains unknown which mechanisms control formation of Z-DNA in the extracellular environment, but Buzzo *et al.* suggested that Z-DNA formation is linked to the presence of Holliday junctions stabilized by DNABII proteins (17). In such DNA protein complexes, the ends of DNA could be locked in a high energy state similar to a supercoiled state conducive for Z-DNA formation.

G-quadruplexes (GQ) are other secondary structures found in G-rich sequences and in bacterial genomes under physiological conditions (29). Typically, three consecutive G-tetrads separated by loops of 1–7 nucleotides are required to form intramolecular GQ (30). Intermolecular GQs may form from two or four single strands of DNA or RNA (31), and higher-order intermolecular GQ-wires can form under molecular crowding conditions (32) or simply in the presence of 10 mM divalent cations (33). Several environmental conditions affect GQ formation. K^+^ and Na^+^ stabilize GQ, while Cs^+^ or Li^+^ do not favor GQ formation (34). Simulations also suggest possible stabilization of GQ by heme, which binds to GQ with high affinity (35). Finally, oxidation of guanines can also affect GQ conformation and stability (36). Notably, GQ motifs are complemented by C-rich sequences that may fold into i-motifs upon cytosine protonation at acidic pH (<5) (37). However, the incidence of i-motifs in bacteria remain unknown.

DNA triplexes are formed by polypurine-polypyrimidine nucleic acid sequences in response to super-coiling (38), polyamines (39), and acidic pH (40), and are highly conserved and abundant in prokaryotic genomes (41).

The genome of strain 1457 is 2 454 929 bp long containing 2260 protein-coding sequences and 81 RNAs with a 32.3% GC content (42). Despite the low GC content, we hypothesize that the availability of specific extracellular polymeric substances, cations, metalloporphyrins, oxidizing conditions, and exposure to mechanical stress that leads to bending DNA to a state similar to supercoiled state could promote formation of Z-DNA and G-quadruplex structures after genomic DNA is released to the extracellular space in biofilms. These conditions are present in *S. epidermidis’* natural environment on the skin, in wound infections and presumably adjacent to implants located in the arteries, bones and the subcutaneous space.

Accordingly, the first aim of this study was to determine if non-canonical DNA structures exist in the matrix of *S. epidermidis* biofilms, and to identify environmental and biological factors that affect formation of these structures. The second aim was to determine if secondary structures are protected from degradation by mammalian DNase I and other nucleases. We use immunolabelling and confocal microscopy to visualize various DNA secondary structures at the μm scale in biofilms formed *in vitro* and *in vivo*, and optical coherence tomography to observe changes in the matrix structure on the μm-mm scale. We furthermore evaluate activity of three nucleases (mammalian DNase I, S1 nuclease and Micrococcal nuclease) against various secondary structures of DNA oligos, and subsequently assess how the properties of these nucleases affect their ability to degrade eDNA in biofilms. Moreover, for the first time we demonstrate extracellular peroxidase activity in biofilms.

## Materials and methods

### Chemicals and buffers

Stock solutions of 1 M Tris hydroxymethyl aminomethane (Tris pH 7.5, Merck), 2 M NaCl (Merck), 2 M KCl (Merck), 2 M MgSO_4_ (Merck), 670 mM CaCl_2_ (Merck) were prepared in milliQ water (18.2 Ω). The pH was adjusted with 33% acetic acid (Merck). A 2% chitosan solution was obtained by dissolving chitosan (high purity, 740063 Merck) in milliQ water with 0.13% (vol/vol) acetic acid followed by repeated cycles of heating to 50°C for 5 minutes and vortexing for 30 s. A 25 mM hemin solution was obtained by dissolving hemin (51280–1G, Merck) in 200 mM Tris with 100 mM NaOH (Merck) and 30% DMSO (pH 11). Hemin was handled and stored away from light. A 1 × GQ-buffer consisted of 10 mM Tris with 100 mM KCl (pH 7.5) and was used for annealing GQ from DNA oligos. A 1 × B-buffer consisted of 25 mM Tris with 6.25 mM CaCl_2_ and 1 mM MgSO_4_ (pH 5.5) and was used for annealing B-DNA and for all DNase treatments. 1 × Z-buffer consisted of 25 mM Tris with 6.25 mM CaCl_2_ and 1 mM MgSO_4_ and 0.025% chitosan (pH 5.5) and was used for annealing Z-DNA. All solutions were sterile filtered using 0.2 μm filters (83.1826.001, Fisher Scientific).

### Fluorescent stains

Picogreen Quant-iT^TM^, TOTO^TM^-1, TOTO^TM^-3, SYTO^TM^60 and FM^TM^4-64 (Thermo Fisher Scientific) were aliquoted to 100 × working concentration in milliQ water. DNA-binding stains were stored at -20°C and FM4-64 was stored at 4°C.

### Synthetic DNA substrates

DNA oligos (Supplementary Table S1) were purchased from Integrated DNA Technologies (IDT) as stocks of 100 μM in 1 mM TE buffer (pH 7.5), purified using standard desalting. Solutions of DNA in either 1 × GQ-buffer (10 mM Tris, 100 mM KCl, pH 7.5) or in 1 × Z-buffer (0.025% chitosan, 25 mM Tris, 6.25 mM CaCl_2_, 1 mM MgSO_4,_ pH 5.5) or 1 × B-buffer (25 mM Tris, 6.25 mM CaCl2, 1 mM MgSO4, pH 5.5) were annealed to obtain pure GQ-proxy, Z-proxy or B-proxy model, respectively. For circular dichroism (CD), 5 μM DNA solutions were annealed to obtain better quality of the signal. For the enzymatic assays, 1 μM DNA solutiuons were annealed.

### Characterization of DNA substrates by circular dichroism

The structure of Z-DNA was verified with the recording of a circular dichroism (CD) spectrum measured on a J-810 Spectropolarimeter with wavelength range at 220–320 nm, data pitch at 0.5 nm, scanning speed at 50 nm/min, response at 4 sec and bandwidth at 2 nm. The measurements were done by loading 60 μl of the sample to a cuvette with 0.3 cm optical path. Samples of 5 μM DNA were prepared in 1 × B-buffer doped with 0–0.1% chitosan and annealed as described previously. The signal measured was CD (mdeg = 0.001 deg), which is related to absorbance by a factor of 32.98. Taking this into account, we converted the CD signal to Δϵ(M^-1^cm^-1^) with use of Lambert-Beer law:


\begin{equation*}\Delta \varepsilon = \frac{{0.001*CD}}{{32.98*c*0.3}}\end{equation*}

The DNA concentrations were calculated in moles of base pairs per liter by multiplying 5 μM concentration by the number of base pairs in the two DNA sequences analyzed.

### Anti-DNA antibodies

The 1 mg/ml antibodies Atto488-BG4 (goat monoclonal IgG lambda), FluoProbes647®-BG4 (goat monoclonal IgG lambda), FluoProbes647®-1H6 (goat monoclonal IgG kappa), Atto488-Z22 (rabbit monoclonal IgG kappa), Atto488-Jel466 (rabbit monoclonal IgG kappa), and Atto488-iMab (rabbit monoclonal IgG kappa) in phosphate buffer saline (PBS) with 0.02% proclin were purchased from the Absolute Antibodies as fluorescently tagged antibodies (subsequently stored in the fridge with light protection). The 1 mg/ml antibody AB1 (mouse monoclonal 35I9) in PBS was purchased from Abcam (aliquoted and stored at −20°C). The 2 mg/ml anti-mouse secondary antibody CF405S-AB2 (goat polyclonal IgG H + L) and untagged 1H6 (mouse monoclonal IgG2bκ) in PBS with 0.05% sodium azide and 50% glycerol were purchased from Merck (stored at −20°C with light protection). We used sterile-filtered 3% bovine serum albumin (BSA heat shock fraction, pH 7, >98%, Merck) in 1× Pierce PBS buffer (Thermo Fisher Scientific) to prevent unspecific immunolabelling.

### Media, strains, cultures and plates for *in vitro* biofilms assays

Autoclaved BHI (Merck, 53286) 37 g/l was used for agar plates (15 g/l agar in BHI) as well as overnight cultures (16–20 h at 37°C with 180 rpm shaking). Autoclaved TSB (Merck, T8907) 30 g/l supplemented with monovalent salts ± hemin was used to obtain biofilms for studying secondary structures of eDNA and their role in biofilm formation. We used the clinical isolates *S. epidermidis* 1457 (WT, Δa*tlE*, and Δ*icaADBC*) (kindly donated by Prof. Holger Rohde, Universitatsklinikum Hamburg-Eppendorf, Hamburg, Germany) and *S. epidermidis* AUH4567 WT (43) strains streaked onto tryptic soy agar (TSA) and kept in the fridge for up to 1 month. *In vitro* biofilms were inoculated from overnight cultures diluted 20 times in TSB into 96-well plates, incubated at 37°C at 150 rpm for 6 h, and subsequently exchanging the media every 20–24 h. The media used was: (i) TSB, (ii) TSB with 5 μM hemin (H-TSB), (ii) TSB with 200 mM NaCl (TSB-NaCl), (iv) TSB with 200 mM NaCl + 5 μM hemin (H-TSB-NaCl), (v) TSB with 200 mM KCl (TSB-KCl), and (vi) TSB with 200 mM KCl + 5 μM hemin (H-TSB-KCl), (vii) TSB with 200 mM CsCl (TSB-CsCl) and (viii) TSB with 200 mM CsCl + 5 μM hemin (H-TSB-CsCl).

Ibidi transparent flat bottom microplate (1.5 polymer coverslip, ibidiTreat tissue culture treated, sterile, square well, Ibidi #89626) was used to grow and treat biofilms for confocal laser scanning microscopy (CLSM). Nunc black flat bottom microplate (Nunclon Delta-Treated, sterile, round well, Thermofisher Scientific 137101) was used as substrate to grow and treat biofilms for optical coherence tomography (OCT).

### Murine osteomyelitis *in vivo* biofilm

This study was approved by the Danish Animal Experiments Inspectorate under permission 2022–15-0201–01133 and was carried out under the supervision of the veterinarians at the Institute of Biomedicine, Aarhus University.

*Staphylococcus aureus* SAU060112, was used for inoculation of the steel implants. An overnight culture was prepared in tryptic soy broth (TSB) media from a single colony of *S. aureus* and incubated for 18 h at 37°C, 180 rpm. The overnight culture was diluted to OD600 = 0.1 (5 × 10^6^ CFU/ml) in fresh TSB and 5 ml were added to falcon tubes with stainless steel implants (Ento Sphinx, Pardubice IV, Czech Republic) and incubated for 18 h at 37°C.

An osteomyelitis infection was established in mice as previously described (44). A total of five 8–10 week old C57bl/6j mice (Janvier Labs, Le Genest-Saint-Isle, France) were included in the study. All animals (*n* = 5) were housed in a IVC (ventilated) cage, at the animal facilities at the Department of Biomedicine, Arhus University at standard room temperature, 12 h day/night cycle with free access to water and food. After an acclimatization period of one week, the mice were sedated with isoflurane inhalation anaesthesia (5% induction and 2% cont.) followed by injection of buprenorphine (0.05 mg/kg s.c.) for analgesia. The left hind leg was shaved and the steel implants were implanted through the cortical part of the tibial bone. The implants were bent in a U-shape and cut as close to the skin as possible, and the skin was then manipulated to cover the implant. Mice were returned to their cages and analgesics (buprenorphine, 0.7 mg/kg) were administered to the drinking water the first four days of infection. Following seven days of infection, animals were euthanized under isoflurane anaesthesia by cervical dislocation, and the implants were removed for CLSM imaging.

### Labelling eDNA using anti-DNA antibodies and fluorescent stains

To reduce unspecific binding, biofilms were treated in 3% BSA in 1 × PBS. After a short blocking step, first, *in vitro* biofilms in separate microwells were incubated with 60 μl solutions of BG4, Z22, Jel466 or iMab antibody (1:100) in the 3% BSA for 75 min. Second, we added 60 μl solution of mouse AB1 antibody (1:100) in the 3% BSA and continued incubation of the biofilms with the two antibodies for another 75 min. Third, after washing the biofilms with 120 μl of the 3% BSA, the biofilms were incubated with 60 μl solution of anti-mouse AB2 antibody (1:150) in the 3% BSA. The biofilms were washed in the 3% BSA and, finally, stained in 10 mg/l solution of FM4-64 in 100 mM NaCl.

The immunostained biofilms were visualized by confocal laser scanning microscopy (CLSM, Zeiss LSM700) equipped with a 63x/NA1.4 Plan-Apochromat objective using 405, 488 and 639 nm excitation (MBS 405/488/555/639). The Atto488-conjugated BG4 and Z22 antibodies were excited by 488 nm laser power 4.5%, and emitted light was collected at < 600 nm (emission at 520 nm at CLARIOstar BMG Labtech plate reader was used for bulk quantification). The CF405S-conjugated AB2 antibody was excited by 405 nm laser power 4.5% and emitted light was collected at < 550 nm (emission at 440 nm at CLARIOstar BMG Labtech plate reader was used for bulk quantification). The FluoProbes 647-conjugated 1H6 and BG4 were excited by 639 nm laser power 4.5% and emitted light was collected at >600 nm. The FM4-64 stain was excited at 488 nm laser power 2%, and emitted light was collected at >600 nm. The detected fluorescence was assigned three colors: blue for CF405S, green for Atto488, red for FluoProbes 647 and red or white for FM4-64. The samples were visualized as three-channel 2D images as well as maximum intensity projections of Z-stacks (3D images). Cells, B-DNA, and non-canonical DNA were quantified as area coverage in 2D images using the software Daime (45).

2.5 μM TOTO-1 was used to stain and quantify total eDNA in *S. epidermidis in vitro* biofilms in the CLARIOstar BMG Labtech plate reader (excitation at 480 nm/emission at 520 nm).

### GQ/hemin peroxidase activity

For probing the GQ-DNAzyme peroxidase-like activity, we used the tyramide signal amplification to deposit fluorophore-conjugated tyramide at the site of activity. Three-day biofilms were grown in H-TSB-NaCl with and without synthetic DNA c-myc-4 (Supplementary Table S1), washed in 100 mM NaCl and subsequently incubated with fluorescent Alexa488-tyramide reagent (B40953, Thermo Fisher Scientific) diluted 1:100 in 1× PBS buffer (pH 7.5) with 0.1% hydrogen peroxide (stock concentration 30%, Merck) and 2 mM ATP (Thermo Fisher Scientific) at room temperature for 1 h. Subsequently, bacterial cells, B-DNA, and GQ were visualized by immunolabelling and FM4-64 staining as described above.

### Optical coherence tomography

We employed OCT to visualize the 3-day *in vitro* biofilms obtained in different media (i) – (viii) (see above) without and with nuclease treatment to provide end-point macroscopic image of the web-like matrix, to identify which media constituents affect the formation of these structures, and whether nucleases can degrade it. The Nunc microwells with the biofilms as well as the spaces between the wells were filled with 100 mM NaCl to the top, and a giant glass coverslip (Caspilor) was placed above the liquid gently to avoid bubble formation. An excess of liquid was removed by a tissue to avoid tilting of the glass. Biofilms were scanned as 500 slices and analyzed using a SD-OCT Ganymede™ 620C1 (Thorlabs GmbH, Lübeck, Germany), with a LSM03 objective lens. Images were acquired with a frequency of 100 kHz and an A-scan averaging of 3. The center of the well (5 mm × 5 mm × 1.49 mm) was imaged with a voxel size of 12 μm × 12 μm × 1.45 μm in the x, y and z dimensions respectively. The biofilm thickness was calculated by a custom written Python script from the two-dimensional B-scans (Supplementary Script). The images were passed through a median filter with a disk-shaped structuring element of radius 2 before image segmentation. The pixels belonging to the biofilm were identified by removing the low-intensity background and high-intensity signal from the plastic substrate by thresholding. The high-intensity pixels of the substrate were used to identify the bottom of the biofilm. The thickness was calculated as the average distance between the top of the biofilm to the plastic substrate, thus including void space within the biofilm.

### Screening nucleases against DNA secondary structures

Three commercial nucleases, i.e. DNase I (4716728001, Merck), S1 (N5661-50KU, Merck), and Micrococcal nuclease (EN0181, Thermofisher) were screened using the pre-fold DNA substrates (Supplementary Table S1) as well as universal custom 1X B-buffer. The protein concentration was optimized to match activity of the three nucleases: 500 u/ml DNase I, 25 u/ml S1 nuclease and 15 u/ml micrococcal nuclease.

We first tested synthetic DNA degradation using 200 nM pure DNA substrates in the in 1 × B-buffer, pH 6, doped with either nuclease or water. The mixtures (180 μl) were assembled directly in Nunc 96-well microwell plate (round, black bottom, untreated, Thermofisher Scientific), mixed, sealed (to avoid evaporation) and incubated at 37°C for 2 h. At the end, 20 μl of the 10 × Picogreen solution or 20 μl of 10 μM SYTO60 was added to each well containing Z-DNA or GQ-DNA, respectively. For comparison, the stains were also added to ds B-DNA and ss DNA treated the same way (with or without nuclease). The synthetic DNA degradation efficiency was then evaluated by quantification of Picogreen fluorescence (ex. 480 nm, em. 520 nm) for ds B-DNA and Z-DNA, or SYTO60 fluorescence (ex. 630 nm, em. 670 nm) for GQ-DNA, ss DNA, and ds B-DNA in a CLARIOstar plate reader (BMG Labtech). Due to variations in fluorescence between samples, we normalized fluorescence against the untreated control samples, i.e. a value of 1 indicates no degradation and a value of 0 indicates full degradation.

The nucleases at the same concentrations (stated above) were used for evaluating nuclease activity against eDNA in biofilms. To investigate eDNA degradation by these nucleases, they were applied directly to the biofilm (grown for 3 days in H-TSB-NaCl at 150 rpm) in the same universal buffer (25 mM Tris buffer with 6.25 mM Ca^2+^ and 1 m Mg^2+^, pH6) and tested at the end-point using CLSM and OCT (see the Materials and methods description above). After 3-hour enzymatic reactions, the biofilms were labelled using OCT and CLSM (following the standard steps of immunolabelling).

## Results

### *S. epidermidis* biofilm form a web-like extracellular matrix in the presence of hemin and NaCl

After confirming that 5 μM hemin did not impede growth (Supplementary Figure S1), we used TSB with 200 mM NaCl and 5 μM hemin to investigate extracellular DNA structures in *S. epidermidis* biofilm from two clinical isolates, strain 1457 and AUH4567.

**Figure 1.**
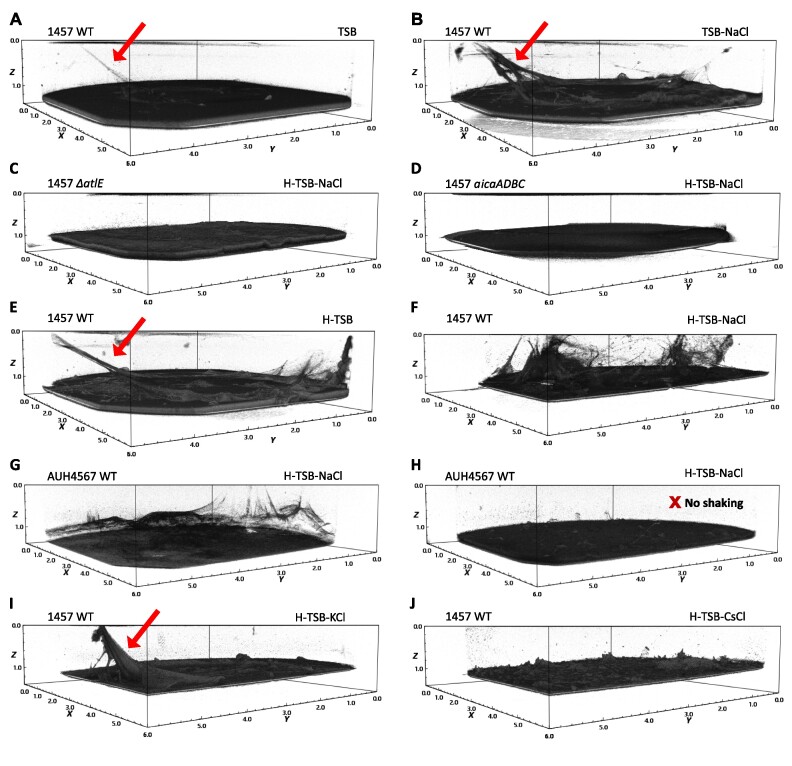
*S. epidermidis* biofilms exposed to mechanical stress produce streamers made from polysaccharides and eDNA. Formation of the streamers relies on the presence of NaCl, KCl and hemin. *S. epidermidis* biofilms were grown for 3 days in TSB with different supplements at 150 rpm rotation or without shaking (H). One biological replicate out of three is shown for **(A)** the 1457 WT in TSB, **(B)** the 1457 WT in TSB-NaCl, **(C)** the 1457 eDNA-deficient mutant Δ*atlE* in the H-TSB-NaCl, **(D)** the 1457 polysaccharide-deficient mutant Δ*icaADBC* in the H-TSB-NaCl, **(E)** the 1457 WT in H-TSB, **(F)** the 1457 WT in H-TSB-KCl, **(G**,**H)** the AUH4567 WT in the H-TSB-NaCl, **(I)** the 1457 WT in H-TSB-KCl, **(J)** the 1457 WT in H-TSB-CsCl. Dimensions are 6 × 6 × 1 mm. Arrows indicate examples of biofilm streamers.

We employed optical coherence tomography (OCT) to visualize biofilms at the micrometer to millimeter scale (46). Here we show that *S. epidermidis* 1457 formed a web-like matrix extending more than 1 mm from the underlying substrate and attaching to the sides of the microwell (Figure 1A, B) in tryptic soy broth supplemented with 200 mM NaCl (TSB-NaCl), resulting in a total concentration of 285 mM NaCl. We hypothesize that these structures are similar to the ‘streamers’ reported for *Pseudomonas aeruginosa* grown under lateral flow (47). The streamers described for *P. aeruginosa* are composed of polysaccharides and eDNA and extend hundreds of μm from the biofilm when subjected to the mechanical stress of liquid flow (47). In our system, the 150 rpm shaking during incubation also creates mechanical stress as the liquid swirls up the sides of the well. To investigate if eDNA and poly-N-acetyl glucosamine (PNAG) contributed to the streamers observed here, we visualized biofilms formed by mutant strains lacking the ability to produce eDNA (1457 *ΔatlE*) (Figure 1C) or polysaccharides (1457 *ΔicaADBC*) (Figure 1D). Neither of these strains produced streamers, suggesting that eDNA and PNAG are essential for their formation.

We hypothesized that formation of streamers under mechanical stress could be linked to the presence of non-canonical secondary DNA structures like Z-DNA and GQ, which are mechanically robust (48) and which increase the viscoelasticity of biofilms (18). We therefore investigated if any environmental conditions that stabilize secondary DNA structures also affected streamer formation. Metalloporphyrins can stabilize Z-DNA (25) and the iron metalloporphyrin hemin binds specifically to GQ (35,49,50). Figure 1E and F shows that addition of 5 μM hemin to the growth media (H-TSB) promotes formation of streamers by *S. epidermidis* 1457. The same effect was observed for the clinical isolate *S. epidermidis* AUH4567 (Figure 1G and Supplementary Figure S2). We also confirmed that streamers only formed under mechanical stress (Figure 1G and H) and at elevated NaCl or KCl (Figure 1I). In contrast, no streamers were seen in the presence of CsCl (Figure 1J), which inhibits GQ.

These analyses did not provide direct evidence for the presence of secondary DNA structures in biofilm streamers. Environmental parameters, such as increased NaCl, can affect the biofilm structure through multiple mechanisms, e.g. by stimulating production of PNAG (51) or impacting the strength of electrostatic interactions between oppositely charged polymeric substances in the biofilm matrix. We therefore proceeded to investigate if the same conditions that led to biofilm streamers actually led to formation of secondary DNA structures in the biofilm matrix by visualizing these DNA structures by immunolabelling.

### *S. epidermidis* biofilm contains eDNA rich in G-quadruplex and Z-DNA

**Figure 2.**
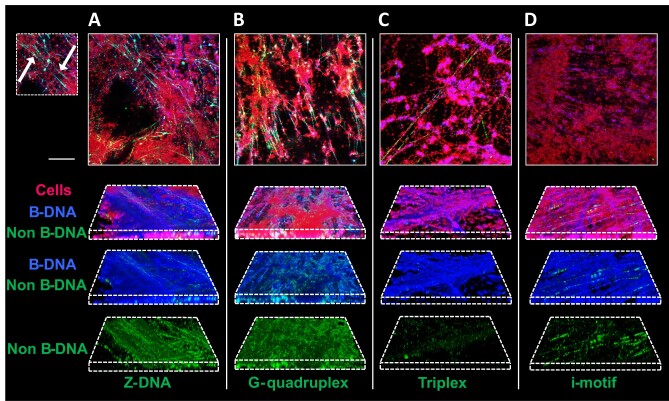
*S. epidermidis* biofilms contain Z-DNA and GQ. *S. epidermidis* AUH4567 biofilms grown for 3 days in H-TSB-NaCl with 150 rpm shaking. CLSM images show bacterial cells in red (FM4-64 stain), B-DNA in blue (AB1-AB2 antibody) and non-B-DNA structures in green (antibodies Z22, BG4, Jel466 and iMab for Z-DNA, GQ, triplex DNA and i-motif, respectively). (**A–D**) CLSM 2D and 3D images of the biofilms, using identical acquisition settings. Scale bar = 20 μm. Arrows indicate examples eDNA strings harboring Z-DNA in (A). Note that 3D images are not from the same position as 2D images.

The next objective was to determine which non-canonical DNA structures were present in the biofilm matrix. The *S. epidermidis* genome has low GC content compared to e.g. *P. aeruginosa* (65–67% GC (52)) that formed GQ-rich biofilm (18) and thus abundance of non-canonical DNA structures in the eDNA of *S. epidermidis* is likely low. We performed a sequence analysis to identify the presence of DNA epitopes mostly used for studies of GQ- and Z-DNA formation and for raising monoclonal antibodies against those structures and found thirty CGCGCG Z-DNA motifs (the ones that have been tested for Z22 antibody) and only a single intramolecular GQ motif GGGCAGGGCAAGTTGGGGTTCGGG. After we confirmed the specificity of the immunolabelling of *S. epidermidis* biofilms using the eDNA-deficient strain Δ*atlE* (Supplementary Figure S3), we optimized immunolabelling of biofilms to visualize total DNA and GQ (Supplementary Figures S4 and S5), and extended these studies to the detection of Z-DNA, GQ, triplex DNA or i-motif together with B-DNA detection (Figure 2A–D). A relative quantification of the non-canonical DNA structures was obtained by normalizing the fluorescence from antibodies binding to non-canonical DNA by the fluorescence from antibodies binding to B-DNA within the sample (Supplementary Figure S6). Quantification was based on area coverage in 2D confocal laser scanning microscopy (CLSM) images in DAIME (45) as well as total fluorescence from fluorometric analysis of entire biofilms in 96-well plates. Among the non-canonical DNA structures, we could detect presence of Z-DNA and GQs. Interestingly, CLSM imaging revealed that these structures were primarily present in web-like strings stretching between clusters of bacterial cells (indicated by arrows in Figure 2A). Moreover, GQs were also detected in matrix that was closely associated with the cell surface of *S. epidermidis* (Supplementary Figure S7).

### Polysaccharides, NaCl, hemin and mechanical stress lead to Z-DNA formation while GQ formation only requires NaCl and mechanical stress

**Figure 3.**
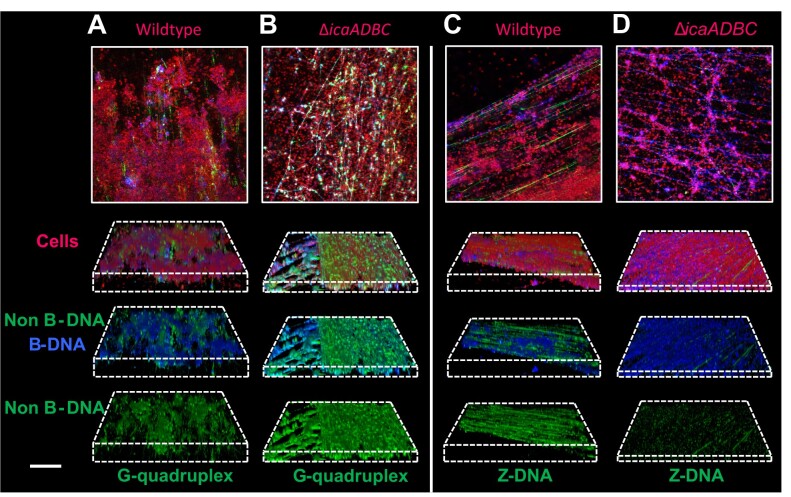
Polysaccharide production stimulates formation of Z-DNA but not GQ. *S. epidermidis* 1457 WT and Δ*icaADBC* mutant strains were grown in H-TSB-NaCl for 3 days (150 rpm shaking). Images show immunolabelling of GQ **(A, B)** and Z-DNA **(C, D)** in *S. epidermidis* 1457 wildtype (A, C) and the isogenic polysaccharide-deficient mutant (B, D). 2D images (top panel) and 3D images (bottom panel) show cells in red (FM4-64 stain), B-DNA in blue (AB1-AB2 antibody), GQ (BG4 antibody) or Z-DNA (Z22 antibody) in green. Acquisition settings are identical in all images (same as in the images in Figure 2). Scale bar = 20 μm.

**Figure 4.**
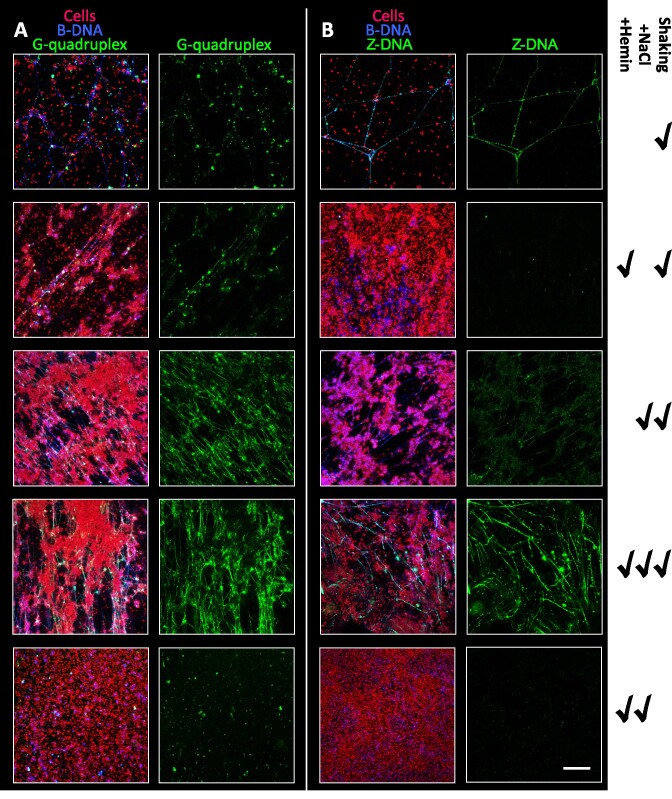
Hemin and NaCl stimulate formation of Z-DNA and NaCl stimulates formation of GQ, but only in combination with mechanical stress from 150 rpm shaking. *S. epidermidis* AUH4567 biofilms were grown for 3 days in TSB with 5 μM hemin or 200 mM NaCl as indicated. **(A)** GQ (BG4 antibody) and **(B)** Z-DNA (Z22 antibody) are shown in green. Cells and B-DNA are shown in red (FM4-64 stain) and blue (AB1-AB2 antibody), respectively. The altering growth conditions are marked by ‘✓’. Scale bar = 20 μm.

The PNAG polysaccharide is abundant in laboratory-grown *S. epidermidis* biofilm (8,53), and it has been shown to associate with eDNA (47), similarly to what has been shown for other cationic polysaccharides in other species (54). We wondered if PNAG played a role in the transition of B-DNA to either GQ or Z-DNA. To address this question, we visualized GQ and Z-DNA formed in biofilms lacking PNAG, using *S. epidermidis* 1457 *ΔicaADBC* (Figure 3). The strain produced significantly thinner biofilm compared to the wildtype strain (Figure 1D, F), and CLSM images revealed abundant GQ (Figure 3A, B), while Z-DNA was almost absent (Figure 3C, D) in the polysaccharide-free biofilms. Interaction of DNA with polycations can transition DNA from B- to Z-form (23), and the partially deacetylated PNAG may thus prompt this transition due to its polycationic nature. To support this hypothesis, we investigated if chitosan, which is similar in structure to fully deacetylated PNAG, could cause the B to Z-DNA transition *in vitro*, using DNA oligonucleotides (Supplementary Table S1) whose conformations were monitored by circular dichroism to distinguish between B- and Z-DNA. Indeed, chitosan caused this transition in a dose-dependent manner, resulting in the full transition to Z-DNA at ≥0.025% chitosan in Tris-acetate buffer at pH 5.5 (Supplementary Figure S8). However, even if PNAG did stimulate the B- to Z- transition through direct interaction with DNA, it could not do it alone: Hemin, NaCl, and mechanical stress were also required (Figure 4B). In contrast, GQ only required NaCl and mechanical stress (Figure 4A).

### Hemin binds to G-quadruplex DNA and forms a peroxidase-like DNAzyme

**Figure 5.**
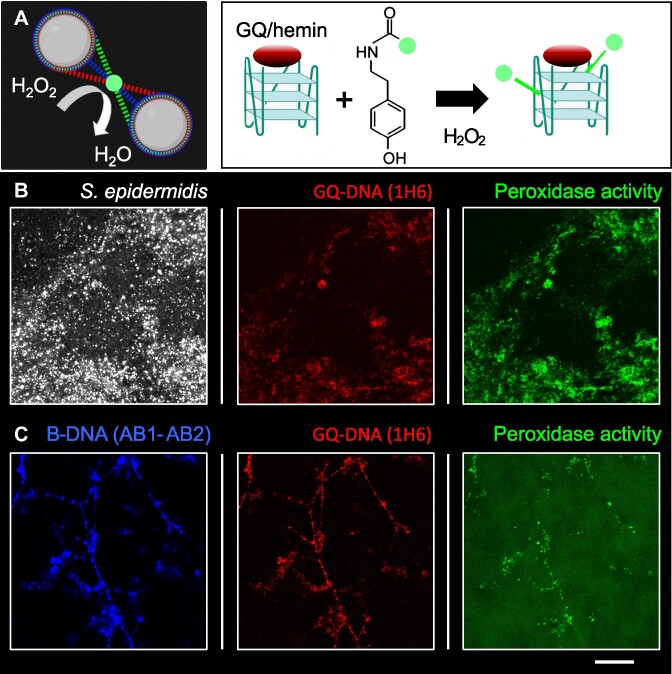
Hemin and GQ enable peroxidase-like DNAzyme activity in biofilms. **(A)** Schematic presentation and **(B)** 3D CLSM and **(C)** 2D CLSM images showing extracellular DNA immunolabelling and tyramide signal amplification (linked to the peroxidase activity) in 3-day *S. epidermidis* AUH 4567 biofilms (H-TSB-NaCl, 150 rpm shaking). (B) Peroxidase (green), GQ (red) and bacteria (white) labelling in biofilms doped with 2 μM c-myc-4. (C) Peroxidase (green), GQ (red) and B-DNA (blue) labelling in biofilms without doping. Scale bar 20 μm.

DNA and RNA G-quadruplexes bind hemin *in vivo* (50) and form a DNAzyme with peroxidase activity similar to horseradish peroxidase (55). GQ/hemin DNAzymes have been studied extensively *in vitro* and used in e.g. biosensing, and so far Lat *et al.* provided a first evidence for GQ-hemin-mediated nucleic acid peroxidase activity to work *in vivo* under the right experimental conditions (55). We therefore visualized the location of DNAzyme activity in *S. epidermidis* AUH4567 biofilms using a reaction similar to tyramide signal amplification (55) followed by immunolabelling of GQ and B-DNA to determine if the peroxidase activity co-localized with GQ (Figure 5A). As a positive control for this method, we grew biofilms supplemented with 2 μM of the GQ-forming DNA oligo c-myc-4 (Supplementary Table S1), and the position of peroxidase activity (green) in these biofilms matched the location of GQ visualized by immunolabelling (red) (Figure 5B). The same co-localization of GQ and peroxidase activity was present in biofilms grown without additional GQ (Figure 5C). While c-myc is known to form a parallel GQ, we expected different types of GQ to form in the biofilms without c-myc. Different variants of GQ vary in peroxidase activity of the GQ/hemin complex, and this could explain why tyramide deposition was not present at all locations with GQ (56). Indeed, the DNAzyme activity was primarily associated with the parallel GQs, and the location of peroxidase activity may thus reflect the location of parallel GQ/hemin complexes (55). We also noticed that the peroxidase activity as well as BG4-immunolabelling was also found in clustered areas that did not overlap with B-DNA immunolabelling, indicating that these G-quadruplexes may be GQ-RNA (19) (Supplementary Figure S9).

### *In vivo Staphylococcus aureus* biofilm from murine osteomyelitis model contains GQ and Z-DNA

**Figure 6.**
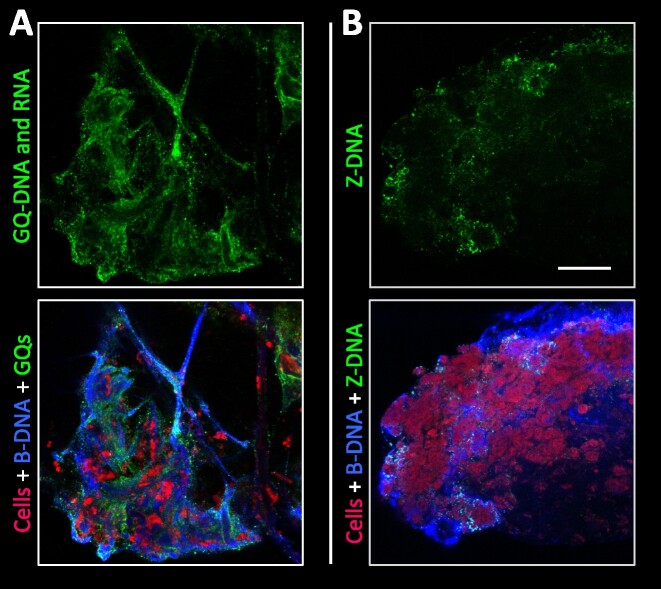
*In vivo* biofilm from murine osteomyelitis model contains GQ and Z-DNA. 2D CLSM images of the tissue attached to tibia implants infected with *S. aureus*: **(A)** GQ and **(B)** Z-DNA. Cells (bacterial and murine) are shown in red (FM4-64 stain), B-DNA in blue (AB1–AB2 antibody) and GQ (BG4 antibody) or Z-DNA (Z22 antibody) in green. The images are shown as single channel (green) as well as three-channel. Scale bar 20 μm.

The formation of non-canonical secondary DNA structures in biofilms may be an observation resulting from the artificial conditions imposed on biofilms grown in the lab. We therefore investigated if GQ and Z-DNA were also present in biofilms grown *in vivo*, where growth of *S. aureus* around an implant in the tibia is in the condition relevant to the mechanical agitation due to movement of the tissue and blood flow around the implant. For this purpose, we used *Staphylococcus aureus* biofilms from a murine osteomyelitis model where 18 h biofilms were formed on the implant in TSB *in vitro*, subsequently inserted into the tibia, and left for 7 days before extraction and CLSM imaging. Figure 6 shows the presence of both GQ and Z-DNA in the tissue attached to the implant surface, confirming that non-canonical DNA structures also form *in vivo*. The biofilm did not have any detectable autofluorescence, however, the implant surface had an autofluorescence in green channel (Supplementary Figure S10). While the infection was not initiated *in vivo*, a previous study by Nishitani *et al.* using a very similar model demonstrated that even though a biofilm is initiated *ex vivo*, by day 7 the biofilm will have undergone substantial changes to distribution and composition in response to the *in vivo* environment (57).

### Micrococcal nuclease degrades GQ-DNA and S1 nuclease has partial activity against Z-DNA

A benefit from forming non-canonical DNA structures in the biofilm matrix could be to protect the biofilm from degradation by host DNases. We therefore investigated if mammalian DNase I or other nucleases could degrade GQ and Z-DNA. To address this question, we developed a fast assay to quantify nuclease activity against GQ-DNA, Z-DNA, ssDNA and B-DNA, and used it to compare double strand-specific DNase I with single strand-specific nucleases of bacteria origin, namely the Micrococcal nuclease originating from *Staphylococcus aureus*, and S1 nuclease originating from *Aspergillus oryzae*. Moreover, micrococcal nuclease is used to degrade total DNA such as chromatin DNA (58) and RNA (59) in sequencing and mapping assays.

**Figure 7.**
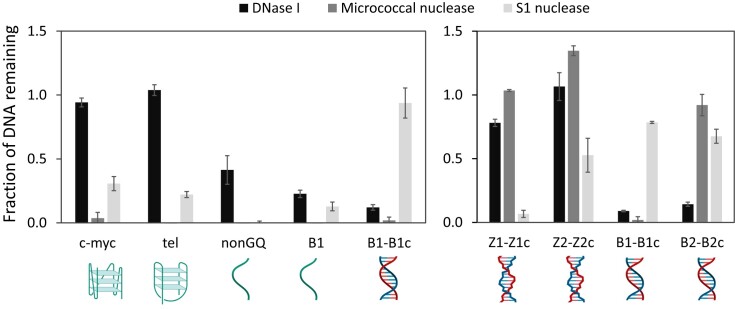
DNase I does not degrade non-canonical DNA structures, while Micrococcal nuclease degrades GQ, and S1 nuclease degrades Z-DNA. Pre-folded oligos (dsDNA, ssDNA, GQ and Z-DNA) were incubated with nucleases for 2 h, and the remaining DNA was quantified and normalized to the untreated control, by quantification of fluorescence from the DNA-binding dye SYTO60 **(A)** or Picogreen **(B)**.

Intra-strand GQ (1 μM) were prepared by folding parallel and hybrid GQ from oligos using c-myc and tel DNA sequences (Supplementary Table S1) in 100 mM KCl. Single-stranded DNA oligos ‘ssB1’ and ‘nonGQ’ (G-rich but unable to form intra-strand GQ) and double-stranded DNA oligos ‘dsB1-B1c’ samples were prepared in the same buffer and included as controls. The B-DNA and non-canonical DNA structures were confirmed by circular dichroism (Supplementary Figure S8). Z-DNA (1 μM) was folded from two complementary DNA sequences in 25 mM Tris buffer, 6.25 mM CaCl_2_ and 1 mM MgSO_4_ (pH 5.5) with 0.025% chitosan. We used a random sequence, ‘Z1’, and a GC-rich sequence, which is assumed to form more stable Z-DNA, ‘Z2’. For comparison, B-DNA of the same sequences were prepared (B1 and B2) without addition of chitosan. The annealed DNA sequences (in their buffers) were diluted to 200 nM in 25 mM Tris buffer, 6.25 mM CaCl_2_ and 1 mM MgSO_4_ (pH 6) to allow optimal nuclease activity before adding the nuclease or water (blanks) to start the incubation. After incubation with nucleases, fluorescent DNA-binding stains were added to assess the amount of undigested DNA remaining (Figure 7). Due to its superior detection of ssDNA and GQ, we used SYTO60 for DNA quantification in samples where GQ and B-DNA were compared (Figure 7A), while Picogreen was used for DNA quantification in samples where Z- and B-DNA were compared (Figure 7B). The ability of nucleases to degrade the different DNA structures was assessed by normalizing the fluorescence intensity after 2 h enzyme treatment to reference samples that did not receive the enzyme, i.e. a value of 1 reflects no DNA degradation, while 0 reflects full DNA degradation. A single sample had a value >1, which may be caused by increased fluorescence of a DNA-binding dye when DNA-binding proteins bind.

Mammalian DNase I degraded dsDNA and ssDNA but had no activity towards GQ and Z-DNA. Micrococcal nuclease fully degraded GQ, ssDNA and dsDNA in the B-DNA form, but had no activity against Z-DNA and GC-rich B-DNA, which shows a sequence bias for this nuclease. Nuclease S1 from *Aspergillus* is known to be specific for ssDNA, and as expected, it fully degraded ssDNA but had no activity against dsDNA in the B-DNA form. However, it partially degraded GQ and Z-DNA, particularly the Z-DNA formed by the random ‘Z1’ sequence. Some activity of S1 nuclease toward these structures was expected, as BZ junctions as well as the single-stranded DNA loops connecting GQ in supercoiled plasmids are susceptible to S1 nuclease (21,60). This notion is consistent with S1 nuclease fully degrading Z-DNA formed by a random DNA sequence, while only partially degrading Z-DNA from GC-rich sequence. It is also possible that these oligonucleotide samples display an equilibrium between B- and Z-DNA, which gives the S1 nuclease an opportunity to digest the DNA during transition events. However, if the activity of S1 nuclease toward Z-DNA can be explained by its ability to cleave ssDNA, we should also have seen activity of Micrococcal nuclease toward Z-DNA, as this nuclease also digests ssDNA. As Micrococcal nuclease has no activity toward Z-DNA, our data indicate that S1 nuclease degrades Z-DNA, but the underlying mechanism and the scope of this activity requires further investigation. In summary, non-canonical DNA structures are resistant to the action of mammalian DNase I, but other nucleases may be used to tackle these robust DNA structures.

**Figure 8.**
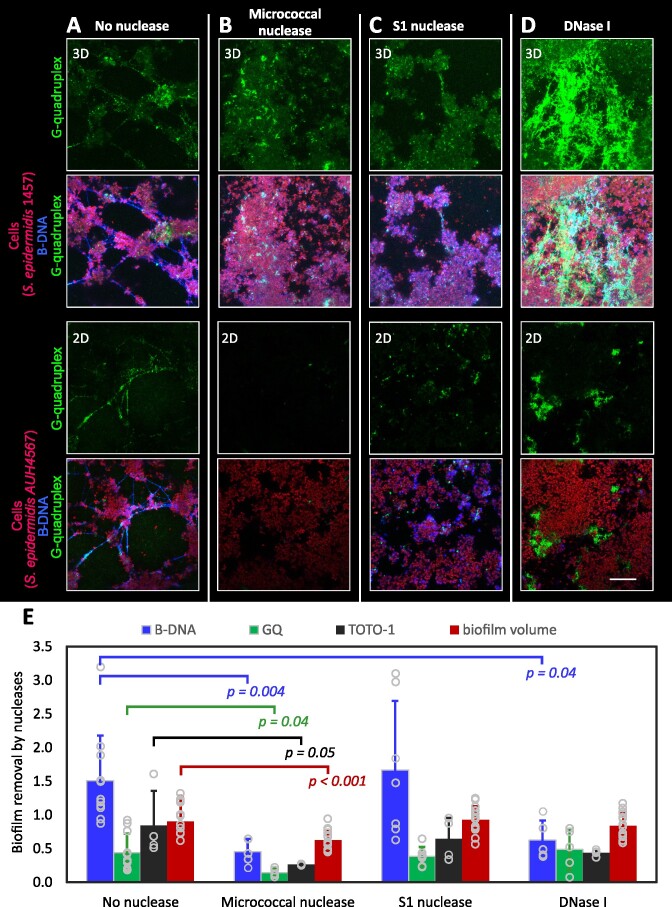
CLSM and OCT imaging of nuclease-treated *S. epidermidis* biofilms show that Micrococcal nuclease is more effective than S1 and DNase I. *S. epidermidis* AUH 4567 and 1457 biofilms were grown in H-TSB-NaCl for 3 days and treated with **(A)** no enzyme, **(B)** Micrococcal nuclease, **(C)** S1 nuclease and **(D)** DNase I in 25 mM Tris-Acetate buffer (pH 6) supplemented with 6.25 mM Ca^2+^ and 1 mM Mg^2+^. Cells are shown in red (FM4-64 stain), B-DNA in blue (AB1-AB2 antibody), GQ (BG4 antibody) or Z-DNA (Z22 antibody) in green. Acquisition settings are identical in all images. Scale bar = 20 μm. **(E)** Quantification of eDNA- and biofilm removal by the nucleases. Analysis of eDNA is based on 2D CLSM analysis of B-DNA (blue bar) or GQ (green bar) normalized to the FM4-64 signal from cells in the CLSM images. Values are mean ± S.D (*n* ≥ 5). Analysis of total eDNA in the biofilms (black bar) based on the bulk TOTO-1 fluorescence measured in the plate reader (*n* = 4) and normalized to the ‘No nuclease’ sample initial fluorescence. Analysis of biofilm volume (mm^3^, red bar) is based on 3D OCT imaging. Values are mean ± S.D (*n* = 15). *P*-values were obtained from symmetrical, two-tailed Student's *t*-test.

Based on the results of pure DNA *in vitro* assays, we hypothesized that Micrococcal nuclease and S1 nuclease could remove GQ in biofilms. To test this, we treated the *S. epidermidis* biofilms in the same buffer and at the same enzyme concentration as used for DNA oligos, and assessed the eDNA remaining in the biofilm by immunolabelling and CLSM while the overall biofilm removal was assessed by label-free 3D imaging using OCT. CLSM images revealed an overall decrease in eDNA in samples treated with the Micrococcal nuclease (Figure 8A,B), indicating removal of both B-DNA and GQ by this enzyme. In contrast, S1 nuclease had little impact on eDNA in the biofilm (Figure 8A, C), and only showed indication of removing some of the web-like DNA strings. DNase I only removed B-DNA, but quite surprisingly, large areas of aggregated GQ emerged after DNase I treatment (Figure 8A, D), indicating the remaining GQs assembled and thus remodeled the extracellular matrix locally. GQs have a tendency to aggregate, and this appears to happen when released from the rest of the DNA network. The biofilm volume after treatment with Micrococcal nuclease, S1 nuclease and DNase I decreased by 36%, 6% and 15%, respectively, which corroborated observations by CLSM (Figure 8E). Collectively, these results suggest that Micrococcal nuclease is more effective than DNase I for implementation in biofilm control.

## Discussion

Many biofilms contain eDNA comprised of genomic DNA originating from lysed bacteria or from immune cells that respond to an infection (13). Outside the controlled environment of a cell, eDNA exists in a variable chemical environment and will interact with other polymeric substances in the biofilm matrix. These interactions and local conditions can cause DNA to adapt non-canonical secondary structures, which may provide new functions and properties of the biofilm matrix that were previously overlooked. In this study, we show large amounts of GQ and Z-DNA in the extracellular matrix of *S. epidermidis* biofilms grown in laboratory medium supplemented with hemin and NaCl. Although the NaCl concentration used in our study was above physiological levels, we found the same structures in biofilms from implant-associated staphylococcal infections. Furthermore, biofilms experience much higher concentrations of NaCl and KCl e.g. on the skin or in the marine environment. It is thus likely that biofilms in many different environments also contain non-canonical DNA structures.

### Formation of Z-DNA in biofilms

Z-DNA only formed in biofilms supplemented with hemin, and the role of hemin in the B-to-Z-DNA transition is intriguing. We speculate that hemin can react with the eDNA, turning guanines into 8-oxoguanines which favor Z-DNA (28), or that hemin forms a coordination bond with the N7 atoms of guanines as it has been proposed for the B-to-Z transition mediated by Zn porphyrins (25). While there is no evidence of hemin forming coordination bond with N7 atom of guanines, a similar coordination bond has been reported between heme and histidine- and tyrosine-based protein motifs (61), supporting that such coordination is a plausible mechanism for hemin's role in Z-DNA formation. *In vivo*, bacteria have access to hemin in plasma (2–5 μM (62)) or on inflamed mucosal surfaces, as exemplified by the requirement for hemin (≈7.5 μM) by some biofilm-forming pathogens (63). We confirmed that both GQ and Z-DNA were present *in vivo* in implant-associated *S. aureus* biofilms from a murine model (Figure 6), and a previous study of Z-DNA also found large amounts of Z-DNA in *in vivo* biofilms from lung- or middle ear infections as well as in *in vitro* biofilms that were grown with hemin (17).

Z-DNA formation also correlated with the presence of the extracellular polysaccharide PNAG, which is a significant component of the *S. epidermidis* biofilm matrix *in vitro* (64). PNAG’s involvement in Z-DNA formation might be indirect by immobilizing eDNA in the biofilm while other circumstances cause the transition from B- to Z-DNA. It is also possible that it directly drives the B- to Z transition due to its polycationic nature. PNAG in *S. epidermidis* biofilms is approximately 15–20% deacetylated (65) which results in a net positive charge at neutral pH. Polycations can promote B to Z-DNA transition (23), which we confirmed in a model system by annealing a normal DNA sequence B1-B1c as well as polyGC DNA in the presence of chitosan as the polycationic agent (Supplementary Figure S8). Bacteria produce several different polycationic matrix components. These include e.g. other positively charged polysaccharides, such as Pel in *Pseudomonas aeruginosa* which also co-localizes with eDNA (54), or polycationic peptides, such as the α-type phenol-soluble modulins which also complexes with eDNA in biofilms (66). It is thus possible that PNAG and other polycationic matrix components contribute to Z-DNA formation in biofilms, but even if they do promote Z-DNA formation, they cannot accomplish this feat alone. We showed that in addition to the right biological and chemical environment in the biofilm matrix, some form of mechanical agitation was also needed for the B- to Z-DNA transition to take place. A driving factor for formation of non-canonical DNA structures might be unzipping of B-DNA due to bending (48). Furthermore, the mechanical stress caused by agitation may promote secondary structures by stretching the DNA, as both tension and torsion can promote B- to Z-DNA transition (67). In addition to the environmental factors and biological components discussed here, Buzzo *et al.* also confirmed the involvement of DNA-binding proteins of the DNABII family, which are universally present in bacteria and are believed to promote Z-DNA formation by bending DNA and by stabilizing Holliday junctions between DNA strands in the biofilm matrix (17).

### Formation of GQ in biofilms

Similar to Z-DNA, we found that elevated NaCl and the presence of mechanical agitation promoted formation of GQ. However, polysaccharides and hemin were not a requirement. Furthermore, GQ was present in both the web-like DNA network as well as in DNA that was associated with the cell surface of *S. epidermidis* (Supplementary Figure S7).

G-quadruplexes can potentially involve both DNA and RNA and can either be intra-stranded (one strand) and inter-stranded (2 or 4 strands) in parallel, hybrid or anti-parallel conformations. The specific form depends on the variety of environmental conditions, e.g. supercoiling (68), crowding (32), type and concentration of divalent (33) and monovalent (34) cations, and the GC content of nucleic acid itself. The only previous study to report G-quadruplexes in biofilms identified GQ-DNA and GQ-RNA by NMR in extracellular nucleic acids extracted from *P. aeruginosa* biofilms and subsequently detected their presence in biofilms by immunolabelling using the antibody 1H6 (18). While the 1H6 antibody detects primarily GQ-DNA in the parallel conformation (69), the BG4 antibody used in our study detects both GQ-DNA and GQ-RNA and in both parallel and anti-parallel conformation (70). The binding of both 1H6 and BG4 antibodies to the total eDNA revealed 1H6 bound in discrete spots while the signal from BG4 was brighter and more continuous along the web-like DNA strings (Supplementary Figure S5). This difference could signify that GQ in the biofilm is both parallel and antiparallel, and that GQ-RNA is also present. Seviour *et al.* did show that monoribonucleotides and DNA exist in the eDNA gel extracted from *P. aeruginosa biofilms*, and that RNA complexed with DNA was a major contributor to biofilm elasticity (19). It is possible that similar mechanisms occur in *S. epidermidis* biofilms.

### Potential biases from DNA immunolabelling

Our observations and conclusions are based on immunolabelling of eDNA in biofilms using monoclonal antibodies 1H6, BG4, and Z22, and the detected DNA structures may therefore be biased towards the epitopes used for raising these antibodies. For example, Z22 was raised against (GC)n epitopes (71), and might under-represent the amount of Z-DNA in the biofilm if it does not bind to Z-DNA with a different sequence motif. However, Zhang *et al.* recently showed that this antibody does bind to Z-DNA with different sequences (72).

The opposite concern is that Z22 binds to GC-rich regions, even if they are not in the Z-DNA form. In some samples, Z-DNA and GQ-DNA seem to appear in morphologically similar structures of the biofilm, i.e. in the web-like DNA network (Figure 2A,B). This apparent overlap could indicate a cross-reactivity of the antibodies to similar sequence motifs rather than their distinct structural epitopes. However, we do not believe that this is the case, as we find GQ-DNA abundant i biofilms that did not contain Z-DNA. For example, GQ-DNA formed in biofilms of the polysaccharide-deficient strain, while Z-DNA did not (Figure 3), GQ-DNA formed in biofilms that were not supplemented with hemin, while Z-DNA did not (Figure 4), and GQ-DNA formed in DNA that was closely associated with individual cells (Supplementary Figure S7), while Z-DNA was only present in web-like structures connecting groups of cells. The Z22 and BG4 antibodies therefore bind to different DNA structures in the biofilm, although both structures are promoted in G-rich regions of the DNA.

It is noteworthy that in many images GQ- and Z-DNA appear to be abundant in the DNA network that connects cells in the biofilm. This could indicate that the structures form under similar conditions, e.g. when mechanical stress is applied to the DNA, or that there is an equilibrium between the structures that are both prone to form in G-rich sequences. Whether this is the case, should be addressed in future studies.

### Benefits from non-canonical DNA structures in biofilms

A potential benefit from having non-canonical DNA structures in the biofilm matrix is their resilience towards DNase I, and this property might explain the numerous reports of DNase I failing to degrade mature biofilms (14,17). Furthermore, eDNA in biofilms grown at high salinity have been reported to be resistant to degradation by DNase I (16). For the first time, we address the effect of different nucleases on B-DNA and GQ in biofilms after showing different activities to B-DNA, Z-DNA and GQ-DNA by three nucleases (Figure 7). While our results confirmed that DNase I is specific to B-DNA, we also discovered that DNase I treatment resulted in formation of large GQ aggregates in the biofilm, which were not observed prior to DNase I treatment (Figure 8). According to the changes in TOTO-1 fluorescence, the most effective nuclease was Micrococcal nuclease, which reduced the GQ-content as well as the biofilm volume the most (Figure 8).

The efficacy of Micrococcal nuclease toward GQ-DNA and S1 nuclease toward Z-DNA (Figures 7 and 8) may reflect evolution of nucleases that enable biofilm dispersal. DNase I can degrade both double- and single-stranded DNA but has highest affinity for double-stranded DNA and is inefficient at cleaving loops (73). In contrast, both S1 nuclease and Micrococcal nuclease have highest affinity toward single-stranded DNA and cleave single-stranded regions in the loop or at the ends of a hairpin (73). Indeed, the conformational junctions between Z-DNA and B-DNA in supercoiled plasmids are known to be susceptible to the ssDNA-specific S1 nuclease originating from *Aspergillus* (21), and synthetic GQ-DNA has been reported to be degraded by Micrococcal nuclease originating from *Staphylococcus aureus* (74). We note that being primarily a ssDNA-specific nuclease, the Micrococcal nuclease did not degrade dsB2-B2c (100% GC).

In addition to protecting biofilms from host nucleases, our results also indicate that GQ- and Z-DNA is involved in formation of the macroscopic streamers. These streamers did not form in biofilms grown without agitation, NaCl, polysaccharides, or DNA. Addition of CsCl, which inhibits formation of secondary structures also inhibited streamer formation. While our evidence for secondary DNA structures in biofilm streamers is indirect, our findings are in line with previous studies showing that streamers consist of DNA and polysaccharides (47) and are also in line with the knowledge that Z-DNA and GQ-DNA increases the mechanical robustness and viscoelastic properties of biofilms (17,18).

Another benefit of GQ-DNA may be linked to their ability to form a DNAzyme. We visualized DNAzyme activity in a biofilm using tyramide signal amplification for the first time (Figure 5). This enabled us to pinpoint the location of extracellular peroxidase activity and correlate its location with that of GQ-DNA without interference from the intracellular enzymatic peroxidases that all aerobic bacteria contain. Peroxidases are usually considered hazardous to bacteria and are natural antimicrobial components of milk, saliva, and released by immune cells, and are used as antimicrobial components in healthcare and food products (75). So how might biofilms benefit from having peroxidases immobilized throughout the extracellular matrix? One benefit might be protection against certain antimicrobials. Peroxidases can break thioether bonds (76) in common antimicrobial peptides, such as nisin, and thereby contribute to the biofilm's resilience.

The ability of GQ-DNA for binding hemin may also play a role in extracellular electron transport. Saunders *et al.* recently showed that *P. aeruginosa* uses eDNA in the biofilm to transport electrons extracellularly from anaerobic to aerobic parts of the biofilm (77). They use the DNA-binding redox-active molecule pyocyanin to mediate the transfer of electrons to DNA. Hemin is also a DNA-binding redox-active molecule if GQ structures are present in the extracellular network of DNA in the biofilm. It would therefore be highly interesting to investigate if GQ-DNA and hemin offers a generic mechanism to achieve extracellular electron transport in a similar way as the pyocyanin/eDNA system of *P. aeruginosa* biofilms.

## Conclusion

In conclusion, we revealed that *S. epidermidis* produces a thick biofilm matrix that contains web-like structures of eDNA with two non-canonical secondary structures: G-quadruplexes and Z-DNA. Formation of these structures is promoted in the presence of salts (Na^+^ and K^+^) that stabilize the structures, and also in the presence of the metalloporphyrin hemin. Furthermore, mechanical stress that pulls at the eDNA is also involved in their formation.

The potential advantages for forming non-canonical DNA structures in the biofilm matrix includes increased mechanical strength and protection against degradation by certain nucleases. Furthermore, GQ/hemin complexes provide sites of peroxidase activity in the extracellular matrix and represent a novel emergent property of the biofilm matrix with many potential biological functions.

## Supplementary Material

gkae034_Supplemental_File

## Data Availability

The data underlying this article will be shared on reasonable request to the corresponding author.
