# Supplementary material for: Extracellular G-quadruplexes and Z-DNA protect biofilms from DNase I, and G-quadruplexes form a DNAzyme with peroxidase activity
Source: Nucleic Acids Res. 2024 Jan 31;52(4):1575–90. doi: 10.1093/nar/gkae034 (PMC10939358; doi:10.1093/nar/gkae034)
Supplement: gkae034_Supplemental_File [file gkae034_supplemental_file.pdf]

ELECTRONIC SUPPLEMENTARY INFORMATION

S. epidermidis tolerates addition of 5 µM hemin into growth media

We investigated whether hemin had an impact onto planktonic growth of *S. epidermidis* AUH4567 in the TSB media (Figure S1). Under the same growth conditions (37°C, no shaking), hemin had slightly attenuated the planktonic growth at its "working" concentration of 5 µM, but it has not reduced the final OD (compared to hemin-free media). However, this slight negative impact of hemin was yet smaller compared to the impact of 5 µM N-methyl-protoporphyrin or 5 µM $FeSO_4$.

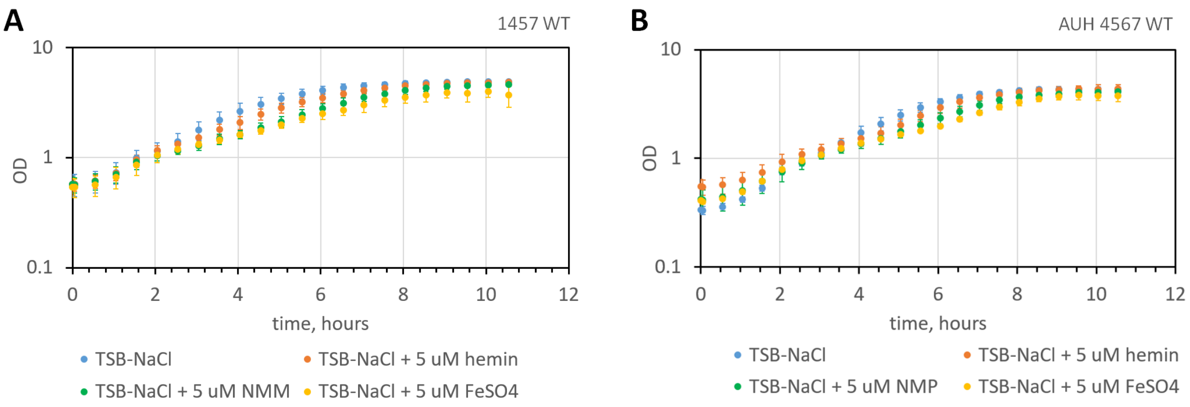

Figure S1. *S. epidermidis* planktonic cultures grow to OD ≈ 5 in TSB-NaCl as well as H-TSB-NaCl. Growth curves of *S. epidermidis* 1457 (A) and AUH 4567 (B) in TSB media amended with 5 µM hemin, 5 µM NMP or 5 µM $FeSO_4$ at 37°C (aerobic with no shaking). Error bars show standard deviations of the mean (N = 6).

S. epidermidis biofilm form a web-like extracellular matrix in the presence of hemin and NaCl

Figure S2 illustrates the web-like biofilm matrix obtained in 3 biological replicates of *S. epidermidis* AUH4567 wildtype in TSB-NaCl vs. H-TSB-NaCl (3-day, 150 rpm shaking) media. Thus, addition of 5 µM hemin to the growth media promoted formation of streamers by both *S. epidermidis* 1457 as well as AUH4567 grown in TSB with 200 mM NaCl (H-TSB-NaCl).

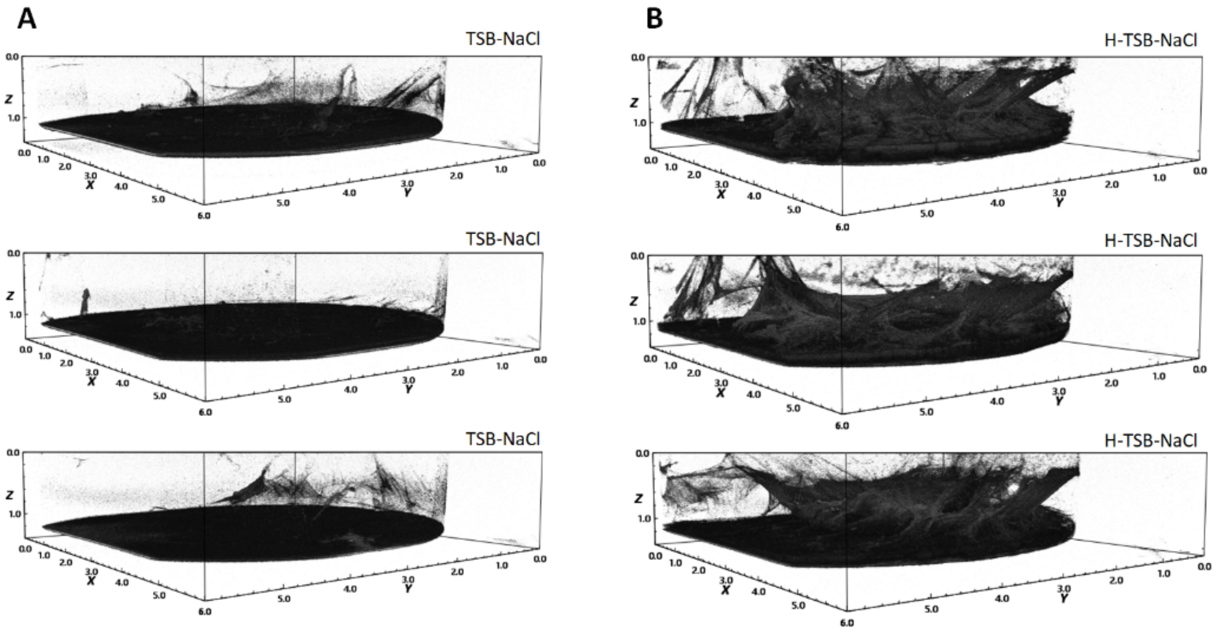

Figure S2. Hemin induced streamers in 3 days old *S. epidermidis* AUH4567 biofilms. Three replicates are shown for (A) the WT in TSB-NaCl and (B) the WT in H-TSB-NaCl. Dimensions are 6 x 6 x 1 mm. See Figure 1 in the main manuscript for *S. epidermidis* 1457 biofilms.

S. epidermidis ΔAtlE mutant deficient in eDNA is producing flat biofilm.

We link formation of the macroscale web-like matrix to eDNA existing in canonical and non-canonical forms detected by confocal laser-scanning microscopy. Figure S3 shows control biofilm imaging of *S. epidermidis* Δ*atlE* mutant deficient in eDNA. Very little or no eDNA was detected in the biofilms from the Δ*atlE* mutants confirming that the antibodies Z22, BG4, and AB1-AB2 did not bind non-nucleic acid component of the exopolymeric matrix (Figure S3A). Furthermore, the deficiency of eDNA explained the lack of macroscale web-like matrix in Δ*atlE* biofilms (Figure S3B).

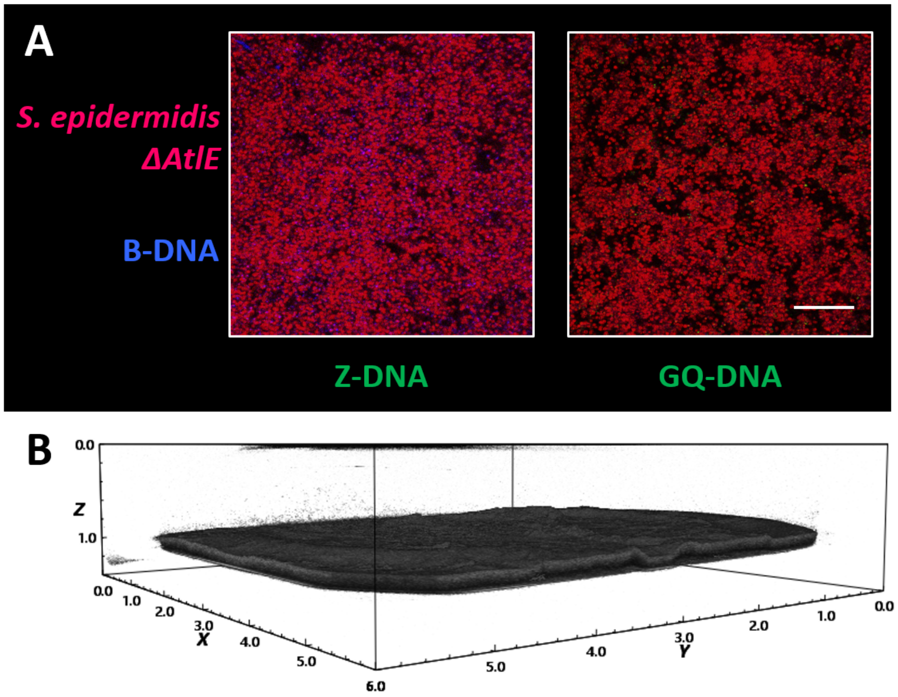

Figure S3. Control imaging of *S. epidermidis* Δ*atlE* biofilm deficient in eDNA revealed lack of antibody binding as well as lack of the web-like extracellular matrix. *S. epidermidis* Δ*atlE* biofilms grown for 3 days in H-TSB-NaCl with 150 rpm shaking. (A) 2D CLSM images show bacterial cells in red (FM4-64 stain), B-DNA in blue (AB1-AB2 antibody) and non-B-DNA structures in green (antibodies Z22 and BG4 for Z-DNA and GQ, respectively). Scale bar = 20 µm. (B) 3D macroscopic image reconstructed using optical coherence tomography. Dimensions are 6 x 6 x 1 mm.

B-DNA is best visualized by immunolabelling compared to using DNA-binding dyes

Using bulk immunofluorescent analysis we demonstrate that DNA binding stain TOTO-3 compromised efficiency of the eDNA immunolabelling by AB1-AB2 antibodies. In this technique, we detect the whole fluorescence of the immobilized antibody in a plate reader. The AB1 binding affinity to the total eDNA in *S. epidermidis* AUH4567 biofilm (3-day in H-TSB-NaCl, 150 rpm) was possibly reduced in biofilms pre-treated in 2.5 µM TOTO-3 compared to untreated biofilms (Figure S4).

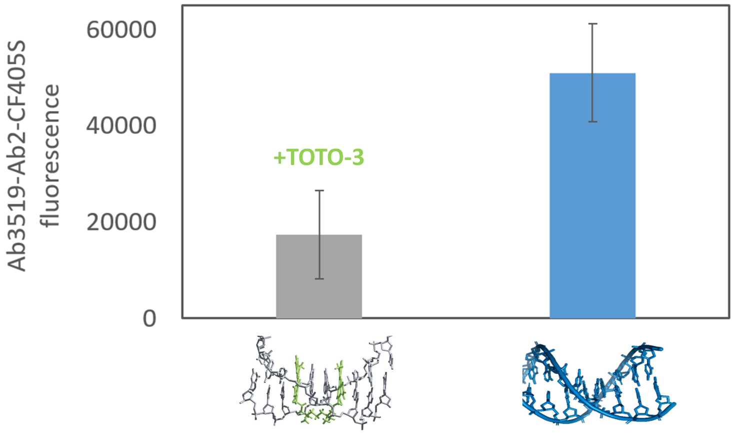

Figure S4. **TOTO-3 reduced efficiency of immunolabelling by AB1-AB2.** Immunolabelling of B-DNA in *S. epidermidis* AUH4567 biofilm (3-day in H-TSB-NaCl, 150 rpm) by the AB1-AB2 with and without prior exposure of the biofilm to TOTO-3. Quantification of the AB2 bulk fluorescence (ex. 405 nm, em. 440 nm) (n=3).

Moreover, we compared the use of a DNA-binding dye (TOTO-3) to immunolabelling of B-DNA (Figure S5 A, B vs. Figure S5 C, D). It was more challenging to visualize the web-like DNA strings by TOTO-3 staining because the signal intensity from DNA strings was very low compared to other areas of the biofilm with highly concentrated eDNA. The strings could therefore easily be overlooked when imaging eDNA with DNA-binding dyes, as acquisition settings are adjusted to visualize the brightest area of the image. In contrast, the signal intensity from B-DNA visualized by immunolabelling was more homogenous. We therefore chose to visualize B-DNA by immunolabelling in the subsequent experiments.

S. epidermidis **biofilm contains extracellular G-quadruplex and Z-DNA**

To validate the abundance of GQs in *S. epidermidis* biofilms, we compared the result from two different GQ-binding antibodies: 1H6 specific to GQ-DNA (Figure S5 A, C) and BG4 specific to GQ-DNA and GQ-RNA (Figure S5 B, D). CLSM imaging revealed minor differences in the distribution of GQ-DNA identified by the two antibodies. The localization of both 1H6 and BG4 antibodies overlapped with the signal from total DNA, indicating that the antibodies did not bind to non-nucleic acid components in the biofilm. However, 1H6 bound in discrete spots while the signal from BG4 was brighter and more continuous along the web-like DNA strings. This difference may reflect the higher affinity of BG4 to intermolecular GQ, or perhaps the presence of GQ-RNA and DNA-RNA hybrids in the web-like structures, as BG4 binds to both GQ-DNA and GQ-RNA while 1H6 only binds to GQ-DNA.

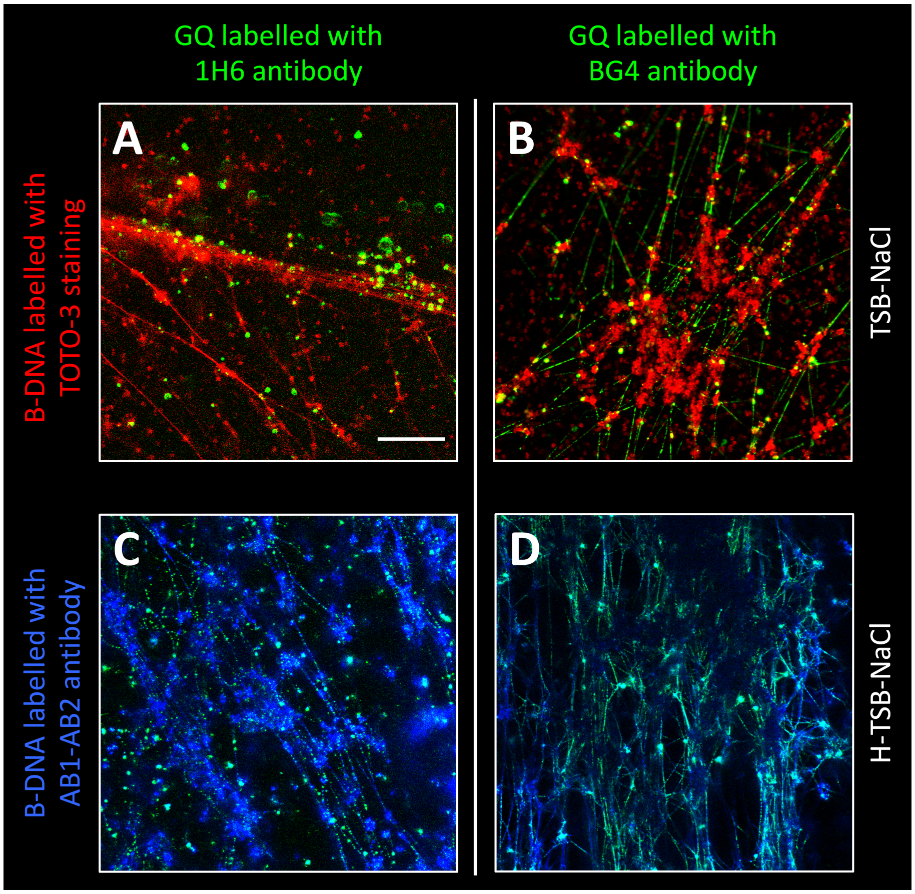

Figure S5. Immunolabelling of GQ by 1H6 and BG4 antibodies shows stronger signal from BG4. B-DNA is best visualized by immunolabelling compared to using DNA-binding dyes. *S. epidermidis* AUH4567 biofilms were grown for 3 days in TSB-NaCl (A, B) or H-TSB-NaCl (C, D) with 150 rpm shaking. (A, B) 2D CLSM images of GQ visualized by antibody 1H6 (A) and BG4 (B) and total eDNA visualized by TOTO-3. (C, D) 2D CLSM images of GQ visualized by antibody 1H6 (C) and BG4 (D) and total eDNA visualized by two-step AB1-AB2 immunolabelling. Scale bar 20 µm.

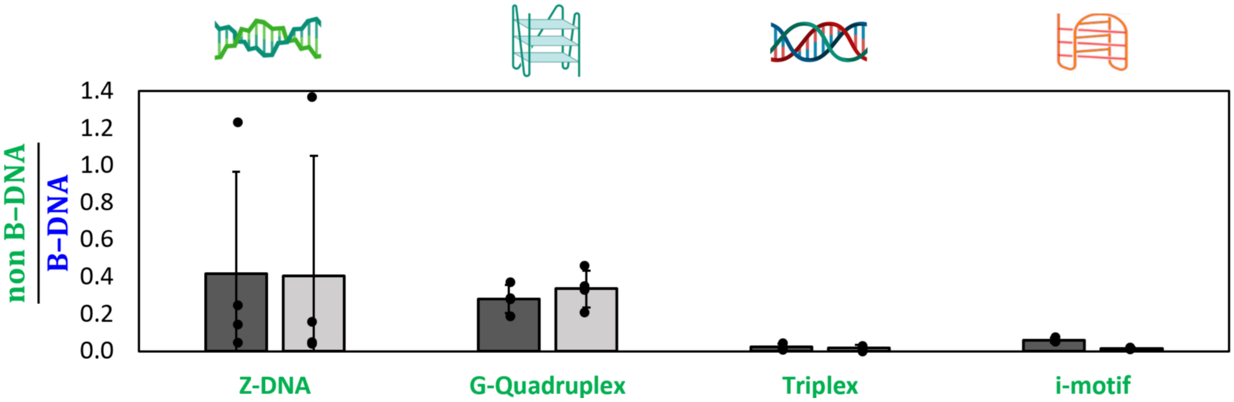

Figure S6. *S. epidermidis* biofilms contain large amounts of Z-DNA and GQ. Quantification of non-B-DNA population relative to B-DNA based on area of fluorescence from 3D CLSM images (light grey bar) and bulk fluorescence from Atto488 (immunolabelled non-B-DNA) normalized to bulk fluorescence from CF505S (immunolabelled B-DNA) (dark grey bar). Bars show mean values ± S.D. (n=4).

We assessed the abundance of the immobilized antibodies Z22, BG4, Jel466 and iMab with respect to the AB1-AB2 from CLSM and bulk immunofluorescent analysis (Figure S6). We detected signals from Z22 and BG4 antibodies confirming presence of Z-DNA and GQ, respectively. The abundance of triplex or i-Motif DNA is unlikely based on our analysis but cannot be excluded since stabilities and affinities of these antibodies as well as hindrance to their targeted binding must be taken into consideration.

G-quadruplexes were consistently found in the eDNA strings (Figure S5) as well as eDNA tightly bound to bacterial membranes (Figure S7). We investigated spatial distribution of BG4 and Z22 antibodies upon their simultaneous application to *S. epidermidis* biofilms. In biofilms, doped with 5µM exogenous GQ-DNA [GA **GGG** T **GGG** TA **GGG** T **GGG**]$_4$, BG4 signal concentrated in the locations around bacteria (Figure S7 A and B), while Z22 signal was detected exclusively in the eDNA string (Figure S7 B). Furthermore, the same distribution of BG4 and Z22 in the vicinity of the cells and in the eDNA strings, respectively, was detected in *S. epidermidis* biofilms without GQ-DNA amendment (Figure S7 C).

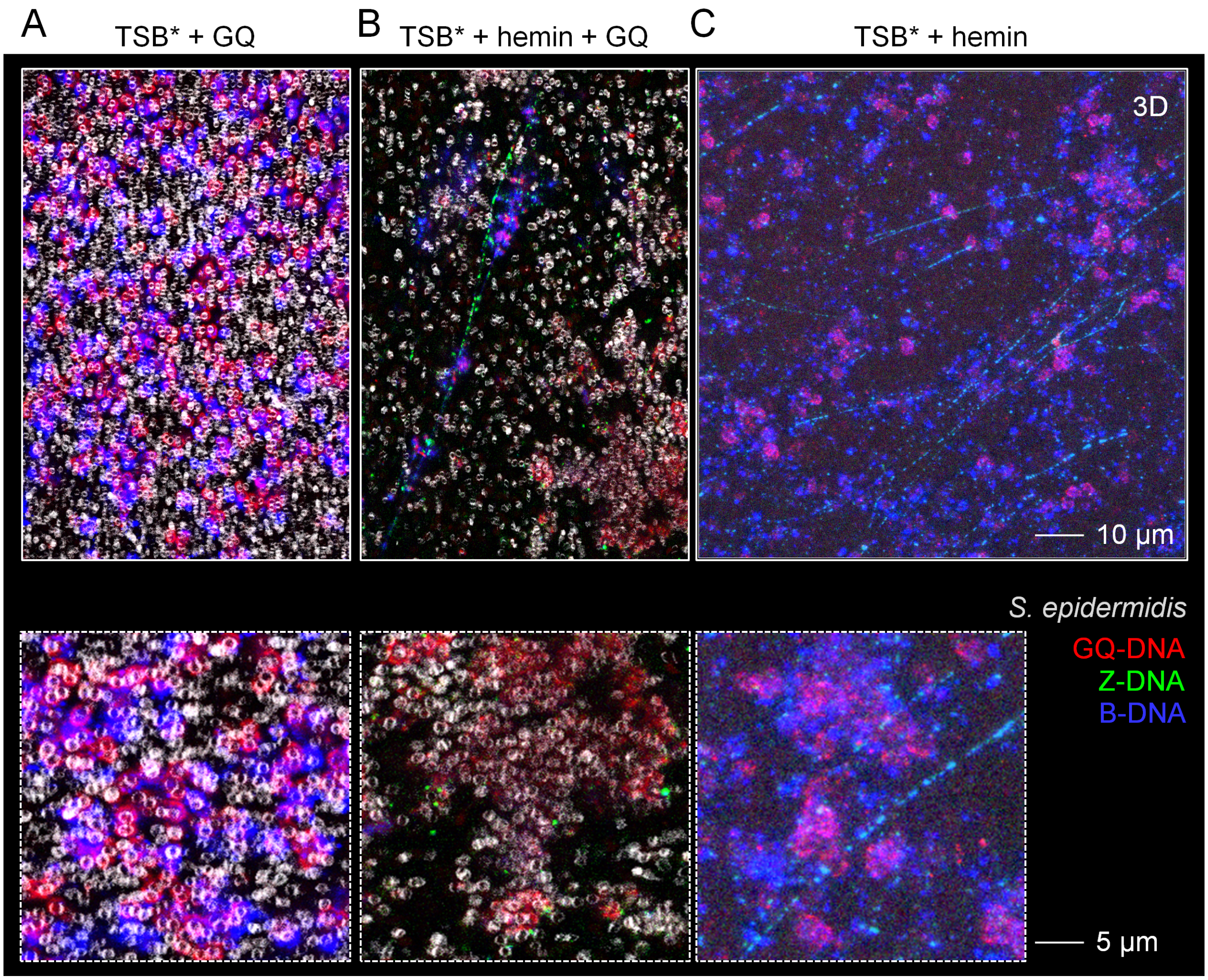

Figure S7. GQs are concentrated around cell surface of *S. epidermidis* while Z-DNA is present in the eDNA strings. *S. epidermidis* AUH4567 biofilms (3 days in TSB*-NaCl with 150 rpm) labelled with BG4 (red), Z22 (green), AB1-AB2 (blue) and FM 4-64 (white). Biofilms were obtained in modified TSB* containing (A) 5 µM synthetic GQ-DNA, (B) 5 µM synthetic GQ-DNA and 5 µM hemin, and (C) 5 µM hemin. (A-B) 2D CLSM, (C) 3D CLSM images.

Polysaccharides and hemin promote formation of Z-DNA in *S. epidermidis* biofilm

As a proxy-model of eDNA, we used synthetic pure DNA sequences (IDT, Table S1) annealed in the buffers optimized for GQ-DNA and Z-DNA in a thermostat.

Table S1. Synthetic DNA molecules used as the substrates for screening enzymes: sequences capable of folding into G-quadruplex DNA (c-myc, c-myc-4 and tel), single-stranded DNA (nonGQ, B1, B2) and their complements (B1c, B2c).

**c-myc**	**GAG GGT GGG TAG GGT GGG**
**c-myc-4**	**GAG GGT GGG TAG GGT GGG GAG GGT GGG TAG GGT GGG GAG GGT GGG TAG GGT GGG GAG GGT GGG TAG GGT GGG CGT CAA CAG ACT CGA**
**Tel**	**TTA GGG TTA GGG TTA GGG TTA GGG TTA**
**nonGQ**	**TAG GGA TGC GAC AGA GAG GAC GGG TA**
**B1**	**GTG GCA GGT CAG TCA AGT ATA CTG CAC TA**
**B1c**	**TAG TGC AGT ATA CTT GAC TGA CCT GCC AC**
**B2**	**GCG CGC GCG CGC GCG CGC GCG C**
**B2c**	**GCG CGC GCG CGC GCG CGC GCG C**

We used circular dichroism to identify DNA conformations formed under conditions either favoring or arresting non-canonical secondary structures. In this work, we for the first time demonstrate flipping B-DNA into Z-form by adding 0.025 % protonated chitosan (pH 5.5) to DNA solutions of B1-B1c (50 % GC) and B2-B2c (100 % GC) prior to their annealing (Figure S8 A, B). Thus, we hypothesize that the partially deacetylated PNAG in a biofilm may prompt this transition due to its polycationic nature.

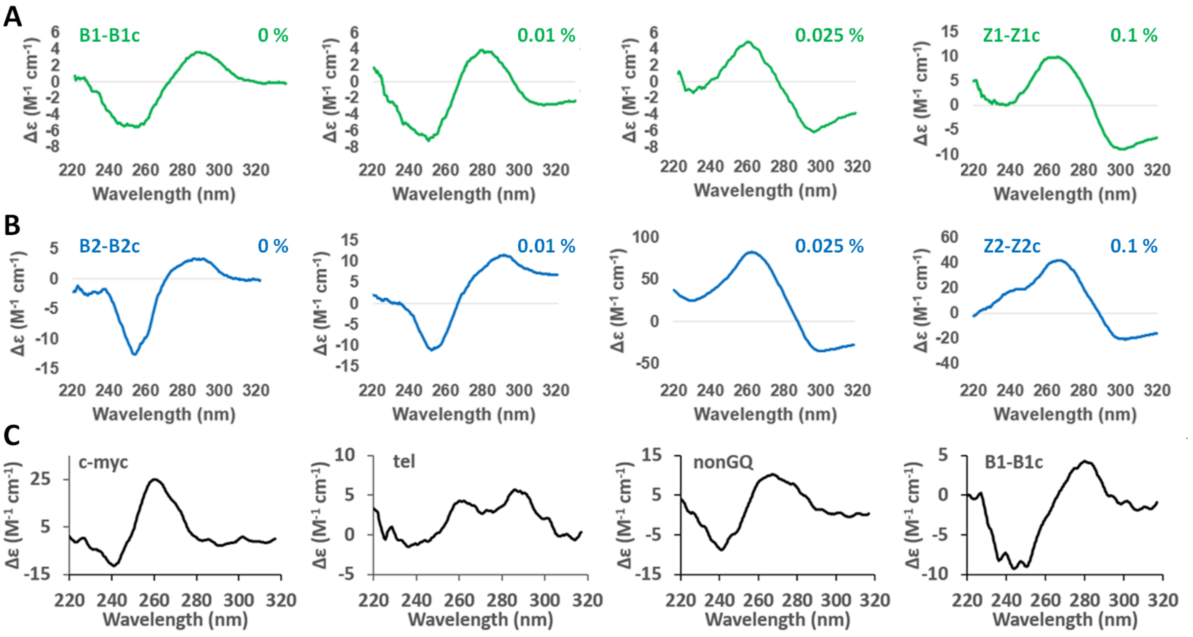

Figure S8. DNA characterization by Circular Dichroism: chitosan flips B-DNA into Z-DNA at pH 6. CD spectra of B-Z-DNA transition (A) between B1-B1c and Z1-Z1c and (B) between B2-B2c and Z2-Z2c as function of chitosan concentration in 25 mM Tris-acetate buffer, 6.25 mM $CaCl_2$, 1 mM $MgSO_4$ (pH 6). Flipping of B-DNA into Z-form was identified as the positive peak shift from 280 nm to 260 nm and at the same time appearance of a negative peak at 300 nm. (C) CD spectra of c-myc, tel, nonGQ and B1-B1c DNA diluted in 25 mM Tris-acetate buffer, 6.25 mM $CaCl_2$, 1 mM $MgSO_4$ (pH 6). The parallel G-quadruplex was identified as the positive peak at 260 nm.

Hemin binds to G-quadruplex DNA in *S. epidermidis* biofilms and forms a peroxidase-like DNAzyme

We demonstrate that GQ-DNA can be used in a biofilm as a DNAzyme with peroxidase activity. This was achieved by adding 0.1 % hydrogen peroxide and fluorescently labelled tyramide to the biofilm without adding further hemin to only detect peroxidase activity associated with hemin trapped in the biofilm. In the GQ/hemin complex, the iron center of hemin chelates hydrogen peroxide, forming an Fe(IV)-O bond and catalyzes the Fenton redox reaction, in which two electrons from a substrate (here tyramide) are transferred to hydrogen peroxide, resulting in water and a chemical bond between the tyramide and amine groups on biomacromolecules in close proximity. This process is also known as tyramide signal amplification due to the ongoing catalysis and local deposition of fluorescent tyramide near the catalyst. G-quadruplex catalyzes this chemical reaction by stabilizing intermediate complexes of the Fenton reaction through the pi-stacking.

In the biofilms that were not doped with c-myc, the peroxidase activity was also detected in non-eDNA strings, supposedly made by GQ-RNA (Figure S9).

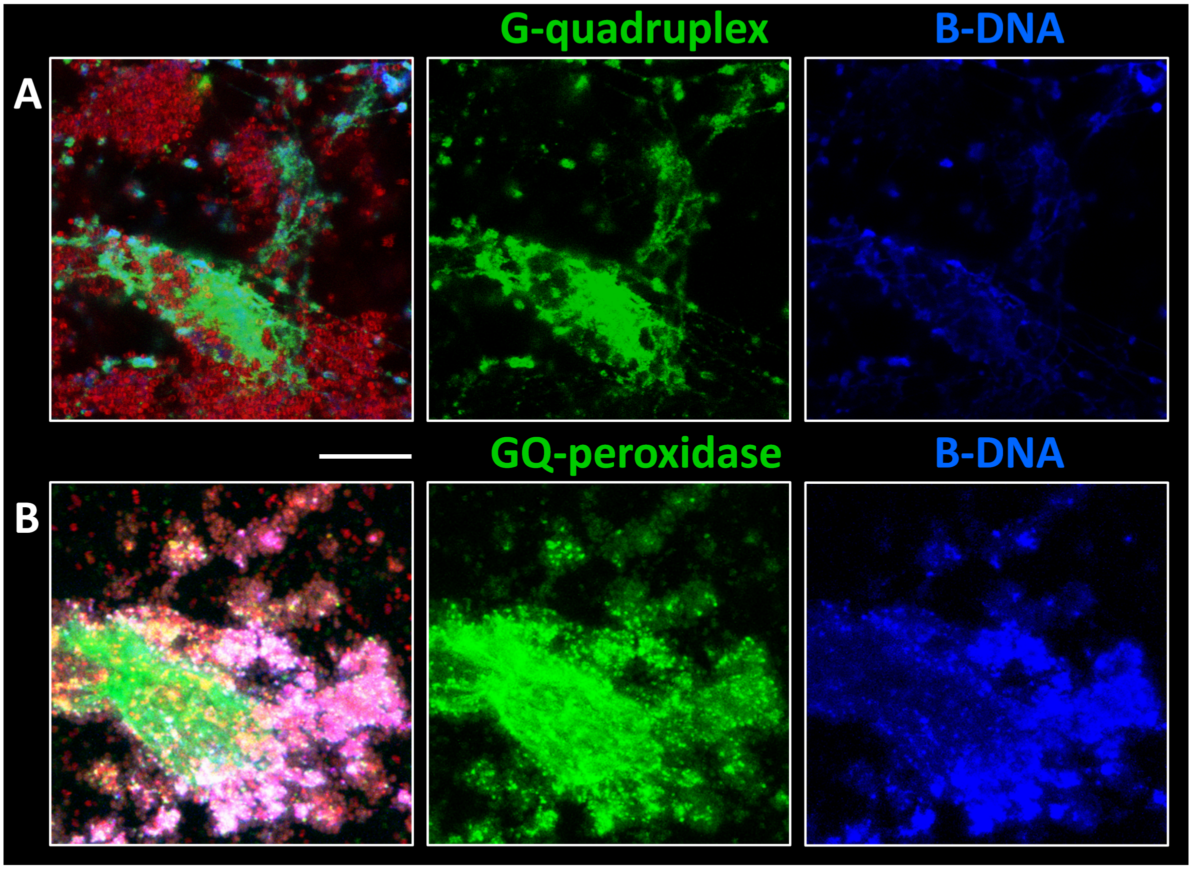

Figure S9. Hemin and GQ-RNA enables peroxidase-like DNAzyme activity in biofilms. *S. epidermidis* AUH 4567 biofilm was grown in H-TSB-NaCl for 3 days. (A) 2D CLSM image of GQ-immunolabelling by BG4 antibody (green) in combination with immunolabelling of B-DNA by AB1-AB2 antibodies (blue) and bacteria by FM 4 64 (red). (B) 3D CLSM image of peroxidase activity by tyramide labelling (green) in combination with immunolabelling of B-DNA by AB1-AB2 antibodies (blue) and bacteria by SYTO60 (red). Scale bar 20 µm.

***In vivo S. aureus* biofilm from murine osteomyelitis model contains GQ and Z-DNA**

The *in vivo* samples were visualized outside the implant area due to the implant autofluorescence in the green channel as well as little biofilm material. Here, the CLSM controls of the autofluorescence as well as further examples of the 7-day biofilms at the implant surface are presented (Figure S10).

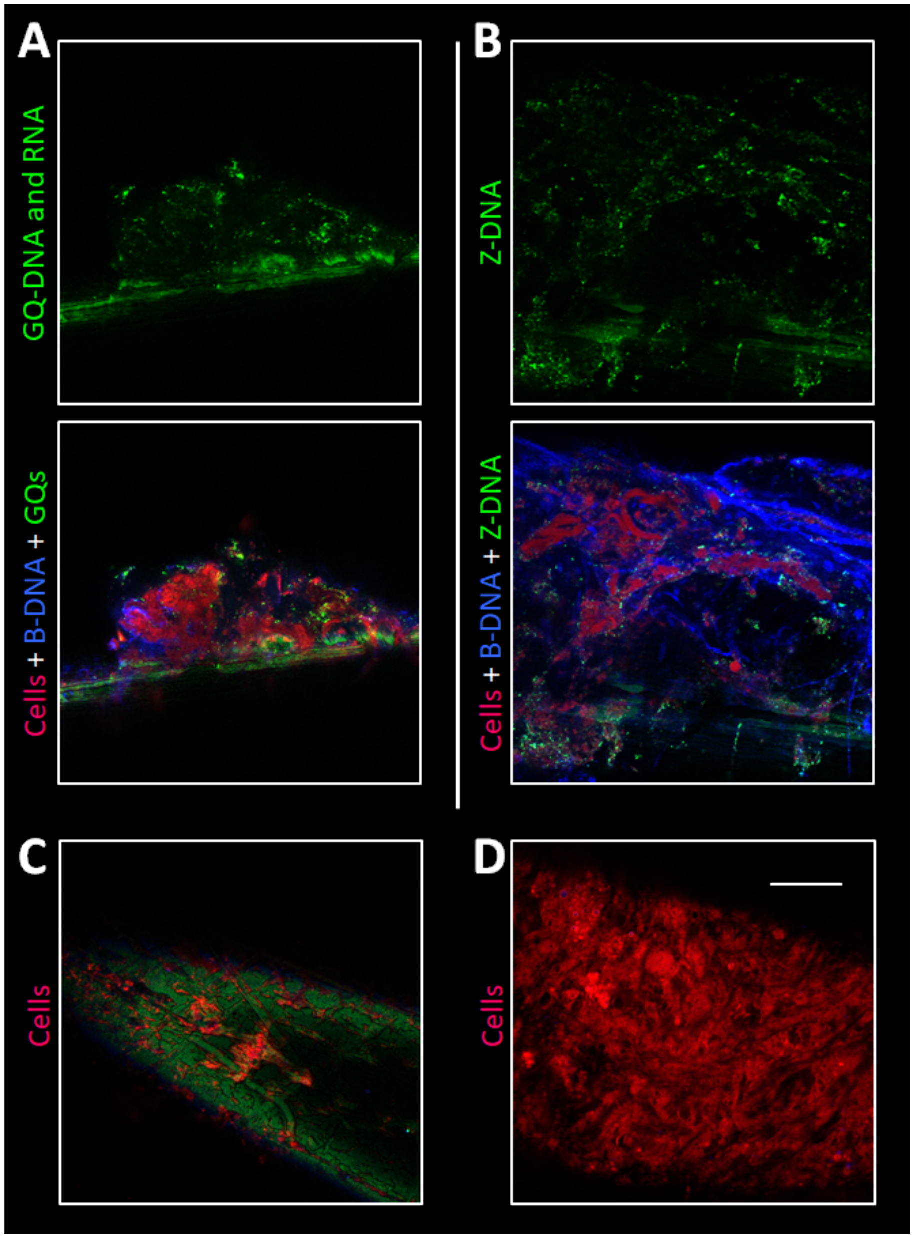

Figure S10. In vivo biofilm from murine osteomyelitis model contains GQ-DNA and Z-DNA. CLSM images of the tissue surrounding tibia implants infected with *S. aureus*: (A) GQ and (B) Z-DNA. (C)-(D) Samples for the autofluorescence controls were stained with FM 4 64. CLSM images were taken directly at the implant surface (C) as well as in the locations in its close vicinity (D). Cells (bacterial and murine) are shown in red (FM4-64 stain), B-DNA in blue (AB1-AB2 antibody), and GQ (BG4 antibody) or Z-DNA (Z22 antibody) in green. The 3D images are shown as single channel (green) as well as three-channel. The biofilms used for autofluorescence imaging contained only FM 4-64 stain. Scale bar 20 µm.
